# Multi-modal transcriptomics: integrating machine learning and convolutional neural networks to identify immune biomarkers in atherosclerosis

**DOI:** 10.3389/fcvm.2024.1397407

**Published:** 2024-11-26

**Authors:** Haiqing Chen, Haotian Lai, Hao Chi, Wei Fan, Jinbang Huang, Shengke Zhang, Chenglu Jiang, Lai Jiang, Qingwen Hu, Xiuben Yan, Yemeng Chen, Jieying Zhang, Guanhu Yang, Bin Liao, Juyi Wan

**Affiliations:** ^1^ School of Clinical Medicine, The Affiliated Hospital, Southwest Medical University, Luzhou, China; ^2^ Metabolic Vascular Diseases Key Laboratory of Sichuan Province, Key Laboratory of Cardiovascular Remodeling and Dysfunction, Department of Cardiovascular Surgery, The Affiliated Hospital, Southwest Medical University, Luzhou, China; ^3^ Key Laboratory of Medical Electrophysiology, Ministry of Education & Medical Electrophysiological Key Laboratory of Sichuan Province, (Collaborative Innovation Center for Prevention of Cardiovascular Diseases), Institute of Cardiovascular Research, Southwest Medical University, Luzhou, China; ^4^ New York College of Traditional Chinese Medicine, Mineola, NY, United States; ^5^ First Teaching Hospital of Tianjin University of Traditional Chinese Medicine, Tianjin University of Traditional Chinese Medicine, Tianjin, China; ^6^ Department of Specialty Medicine, Ohio University, Athens, OH, United States

**Keywords:** atherosclerosis, convolutional neural networks, machine learning, molecular docking, molecular subtyping

## Abstract

**Background:**

Atherosclerosis, a complex chronic vascular disorder with multifactorial etiology, stands as the primary culprit behind consequential cardiovascular events, imposing a substantial societal and economic burden. Nevertheless, our current understanding of its pathogenesis remains imprecise. In this investigation, our objective is to establish computational models elucidating molecular-level markers associated with atherosclerosis. This endeavor involves the integration of advanced machine learning techniques and comprehensive bioinformatics analyses.

**Materials and methods:**

Our analysis incorporated data from three publicly available the Gene Expression Omnibus (GEO) datasets: GSE100927 (104 samples, 30,558 genes), which includes atherosclerotic lesions and control arteries from carotid, femoral, and infra-popliteal arteries of deceased organ donors; GSE43292 (64 samples, 23,307 genes), consisting of paired carotid endarterectomy samples from 32 hypertensive patients, comparing atheroma plaques and intact tissues; and GSE159677 (30,498 single cells, 33,538 genes), examining single-cell transcriptomes of calcified atherosclerotic core plaques and adjacent carotid artery tissues from patients undergoing carotid endarterectomy. Utilizing single-cell sequencing, highly variable atherosclerotic monocyte subpopulations were systematically identified. We analyzed cellular communication patterns with temporal dynamics. The bioinformatics approach Weighted Gene Co—expression Network Analysis (WGCNA) identified key modules, constructing a Protein-Protein Interaction (PPI) network from module-associated genes. Three machine-learning models derived marker genes, formulated through logistic regression and validated via convolutional neural network(CNN) modeling. Subtypes were clustered based on Gene Set Variation Analysis (GSVA) scores, validated through immunoassays.

**Results:**

Three pivotal atherosclerosis-associated genes—CD36, S100A10, CSNK1A1—were unveiled, offering valuable clinical insights. Profiling based on these genes delineated two distinct isoforms: C2 demonstrated potent microbicidal activity, while C1 engaged in inflammation regulation, tissue repair, and immune homeostasis. Molecular docking analyses explored therapeutic potential for Estradiol, Zidovudine, Indinavir, and Dronabinol for clinical applications.

**Conclusion:**

This study introduces three signature genes for atherosclerosis, shaping a novel paradigm for investigating clinical immunological medications. It distinguishes the high biocidal C2 subtype from the inflammation-modulating C1 subtype, utilizing identified signature gene as crucial targets.

## 1 Introduction

Atherosclerosis, characterized as a chronic inflammatory vascular disease with diverse etiologies (1) stems from the intricate interplay among activated endothelial cells, modified low-density lipoprotein (LDL), monocyte-derived macrophages, T-cells, and the vascular wall (2). This pathological process gives rise to atherosclerotic plaques, endothelial dysfunction, inflammation, and plaque formation (3). Notably, a significant epidemiological facet of cardiovascular disease (CVD) is atherosclerotic cardiovascular disease (ASCVD), contributing to a substantial proportion of CVD-related deaths. In 2016 alone, approximately 2.4 million fatalities were attributed to ASCVD, constituting 61% of CVD deaths and 25% of total mortality (4). Premature cardiovascular events resulting from atherosclerosis underscore the urgency of addressing this condition (5). Atherosclerotic alterations increase the risk of premature myocardial infarction, with around 90% of cases traced back to acute thrombus formation leading to arterial blockage at the rupture point of the atherosclerotic plaque (6, 7).

Early stages of atherosclerosis may elude noticeable symptoms (8), emphasizing the critical role of monitoring LDL and ox-LDL changes even before overt symptoms manifest (9) By the time symptoms become apparent, atherosclerosis typically reaches an advanced stage (10). Various clinical approaches exist for evaluating atherosclerosis, including Doppler ultrasound to gauge carotid artery intima-media thickness and detect arterial plaque, as well as dual—source computed tomography of coronary arteries or coronary angiography to assess atherosclerosis presence and quantify coronary artery stenosis (11). However, each diagnostic tool possesses inherent limitations, potentially hindering recognition of atherosclerosis.

Furthermore, the understanding of the mechanism and etiology of atherosclerosis remains a subject of ongoing debate and lacks consensus (12). Divergent perspectives, such as the lipid infiltration theory (13) and the damage-response theory, contribute to the complexity of elucidating its underlying causes. Although vascular inflammation (14), hyperlipidemia (15), and diabetes are widely acknowledged as risk factors, the genesis of atherosclerosis involves a intricate interplay of multiple genes and their products. It is of paramount importance to comprehensively unravel its pathogenesis for early disease diagnosis and intervention.

Given the intricate nature of atherosclerosis, exploring its immune mechanisms and advancing treatment/diagnostic modalities holds significant clinical value (16). This avenue of research is pivotal in enhancing our understanding of the disease and refining strategies for effective diagnosis and intervention.

Moreover, the field of bioinformatics analysis, which integrates computer science and information technology into biological research, has gained increasing prominence in recent decades, spanning diverse domains such as genomics, proteomics, and structural biology. Technological advancements have significantly broadened its scope, exerting a profound impact on comprehending biological systems, investigating disease mechanisms, and facilitating drug development (17). Machine Learning, a subset of Artificial Intelligence (AI), has emerged as a powerful tool for elucidating the intricate relationships between gene expression patterns and diseases (18). Employing sophisticated machine learning and deep learning algorithms allows for a systematic and thorough analysis of vast clinical datasets. This approach facilitates the precise identification of molecular targets and enables accurate assessment of disease risk (19, 20).

Furthermore, the molecular docking technique, a widely employed method, is a notable application for studying molecular interactions, particularly the binding between proteins and small molecules (21). This technique finds application in drug design, predicting binding modes between potential drugs and target proteins to enhance screening efficiency and design novel drug molecules. This not only expedites the drug discovery process but also mitigates experimental costs and time (22). The synergistic integration of bioinformatics, machine learning, and molecular docking techniques holds promise for advancing our understanding of diseases like atherosclerosis and accelerating therapeutic developments.

In light of the global prevalence and substantial impact of atherosclerotic disease, our research endeavors to elucidate the molecular intricacies of atherosclerosis at the single-cell level. We aim to construct an integrated approach, combining transcriptome sequencing and single-cell sequencing technologies. In the realm of single-cell sequencing, our focus is to unravel the nuanced cellular mechanisms underlying atherosclerotic disease progression. This involves a comprehensive exploration of intricate cellular differentiation pathways and communication mechanisms, mirroring the microenvironments influencing gene expression during atherosclerosis progression.

At the transcriptome level, our methodology synergizes machine learning algorithms with a CNN model to identify central genes characterizing atherosclerosis. The performance of this approach is subsequently validated through functional assessments, employing the Receiver Operating Characteristic Curve (ROC). Molecular docking is employed to furnish metrics for predicting clinical drug therapy efficacy against the modeled genes. Additionally, patients with atherosclerosis are categorized into two subtypes, and immune infiltration analysis unveils the immune mechanisms distinguishing these subtypes. This analysis contributes valuable insights into the pathogenesis of atherosclerosis.

We anticipate that our research findings will furnish robust academic support, offering practical applications in the diagnosis, prognosis, and drug therapy of clinical patients dealing with atherosclerosis.

## 2 Method

### 2.1 Raw data processing

**Figure 1:**
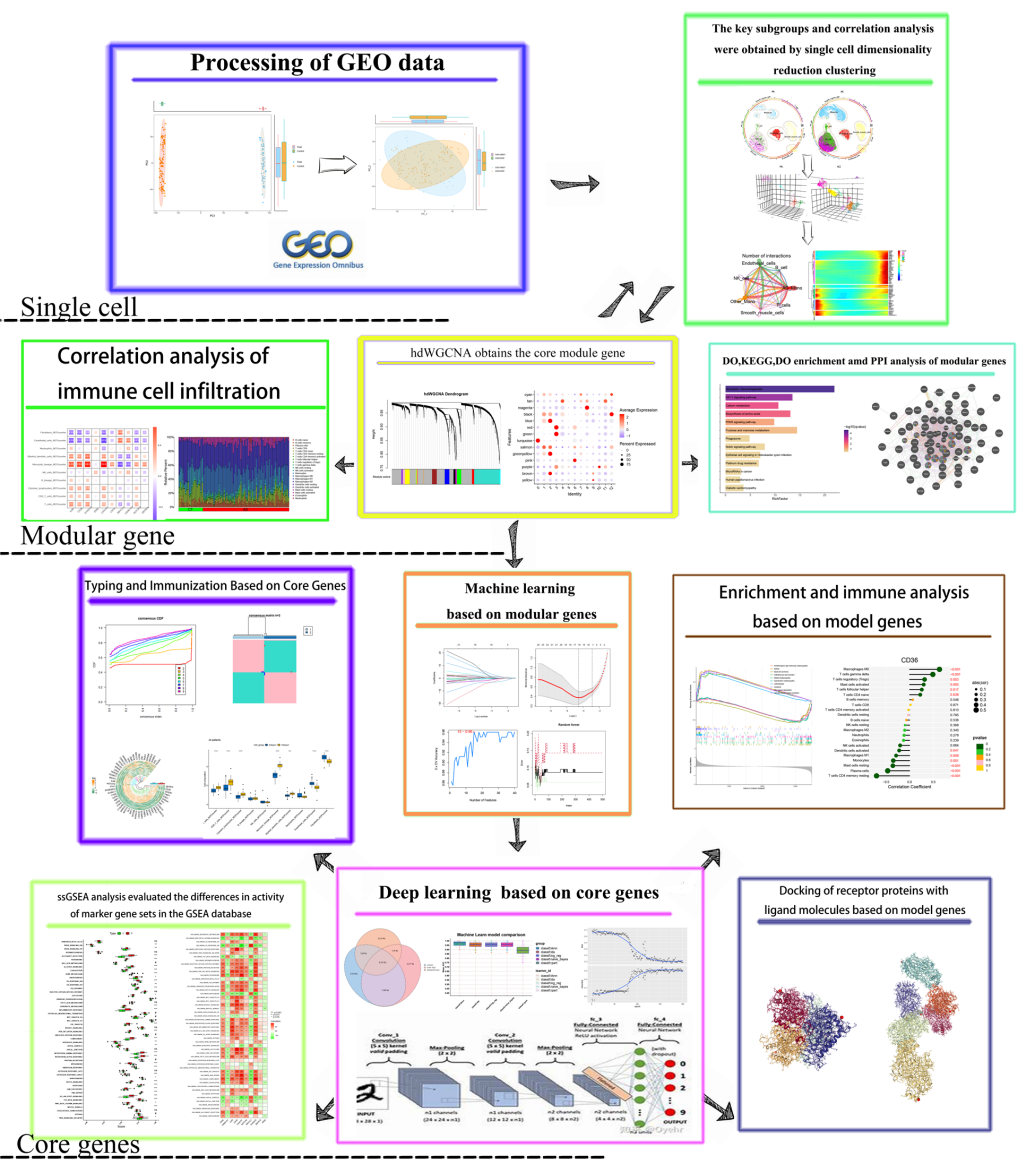
The flowchart shows the research idea of this paper.

The conceptual framework of this study is illustrated in Figure 1. Total RNA data were sourced from the GEO database, specifically the raw array analysis expression datasets GSE100927 (comprising 35 normal samples and 69 disease samples) and GSE43292 (comprising 32 normal samples and 32 disease samples). Model genes were identified through analysis of GSE100927, while diagnostic models were validated using GSE43292. To mitigate batch effects both between and within datasets, the R package “limma” (23) with “normalization between arrays” was applied. The Combat function's efficacy was assessed using principal component analysis (PCA). Subsequently, the probe ID of each gene was mapped to its corresponding Gene symbol. In cases where a Gene symbol corresponded to multiple probe IDs, the average expression of the probe IDs was utilized instead of their individual expression values. Two sets of well-correlated single-cell data were extracted from the GSE159677 dataset. This dataset comprises single-cell RNA-seq data derived from three atherosclerotic core plaques and three patient-matched proximal portions of carotid collaterals, respectively.

### 2.2 scRNA-Seq downscaling to identify key subpopulations

The dimensionality reduction process for the two pairs of single-cell sequencing data is delineated as follows:

(1) Utilize the “Seurat” package within the R software to transform the four single-cell sequencing datasets into Seurat objects (24). (2) Perform quality monitoring to filter out instances with excessively low or high numbers of RNA features and assess the proportion of mitochondrial RNA.(3) Employ the “FindVariableFeatures” function to identify highly variable genes and execute Unique Molecular Identifier (UMI) counting. (4) Normalize the single-cell data using the “Harmony” package (25). (5) Implement Uniform Manifold Approximation and Projection (UMAP) for dimensionality reduction and clustering analysis based on the 2,000 highly variable genes post-normalization (26). (6) Employ the “SingleR” package for cluster annotation to identify cell types (27). (7) Select cell groups exhibiting significant differences and repeat steps 1–5 to iteratively obtain key subgroups.

### 2.3 Cellchat analysis

Cell communication analysis was conducted utilizing the “CellChat” package within the R software environment (28). The createCellChat function was employed to generate a CellChat object, while the identifyOverExpressedGenes function facilitated the identification of over-expressed genes. Subsequently, the identifyOverExpressedInteractions function was utilized to extract ligand-receptor pairs. The projectData function enabled the projection of data onto the PPI network. Furthermore, the createCellChat function was again utilized to create a CellChat object, the identifyOverExpressedGenes function was employed to identify over-expressed genes, and the identifyOverExpressedInteractions function was used to retrieve ligand-receptor pairs. Projecting this data onto the PPI network was accomplished using the projectData function. Finally, the communication probability of the cell was calculated, elucidating inter-cell interactions and communication mechanisms. Functions such as composeCommunProb were instrumental in achieving this outcome.

### 2.4 hdWGCNA (high dimensional weighted gene co-expression network analysis)

High-dimensional weighted gene co-expression network analysis, facilitated by the “hdWGCNA” package, was conducted to probe co-expression modules associated with small subpopulations of monocytes and propose temporal sequence analysis. This method, documented in literature (29–32), was employed to explore the intricate relationships between gene networks, diseases, and clinical features. The analysis involved obtaining metacells through the aggregation of neighboring cells. Sparse matrices of single cells were constructed, and neighbor matrices were generated using TestSoftPowers to establish soft thresholds, subsequently transformed into Topological Overlap Matrix (TOM) matrices. Modules were delineated using hierarchical clustering tree-based techniques. The definition of these modules involved mirror tests employing hierarchical clustering and dynamic tree-cutting. Module attributes of affiliation (MM) and genetic significance (GS) were employed to assess correlations between modules and clinical features. Core co-expression modules, characterized by high module affiliation (MM>0.8) and substantial clinical significance (GS>0.2), were prioritized for association with small subpopulations of single cells. The “Monocle” package was then utilized for cell trajectory reconstruction analysis, employing gene counts and expression to infer cellular differentiation trajectories (33–35). First, to analyze gene dispersion, we used the dispersionTable function. Next, we identified highly variable genes based on criteria of average gene expression ≥0.1 and empirical dispersion exceeding the fitted dispersion by at least one fold.Then, we visualized temporal differentiation pathways using the plot_cell_trajectory function, with color intensity indicating differentiation stages. The diagram demonstrated the progression from top to bottom and from the middle to the sides.Furthermore, to confirm differentiation directions, we utilized an evolutionary tree diagram at the state level. Finally, we analyzed significantly altered genes in the target modules derived from hdWGCNA, observing differentiation patterns from top to bottom and from the middle to the sides.Overall, this approach facilitated the exploration of cellular states and dynamics during biological processes, unveiling insights into the changes and dynamics of cells throughout these processes.

### 2.5 Immune infiltration analysis

The assessment of immune cell composition was carried out utilizing the “CIBERSORT” package (36). Additionally, immune scoring was performed employing the “GSVA” package with immunocompetent gene sets (h.all.v7.5.1.symbols.gmt). The correlation analysis aimed to quantify differences in immune cell numbers, functions, and pathways between atherosclerotic and normal samples. The specific gene sets used were from h.all.v7.5.1.symbols.gmt (37). This comprehensive immune infiltration analysis provided valuable insights into the immune landscape of atherosclerotic samples in comparison to normal samples, shedding light on alterations in immune cell populations, functions, and pathways associated with atherosclerosis.

### 2.6 Module gene enrichment analysis

Enrichment analysis of model genes was conducted employing the Kyoto Encyclopedia of Genes and Genomes (KEGG) and Gene Ontology (GO) using the Differential Ontology (DO) software packages. The benjamin-hocheberg method or False Discovery Rate (FDR) method was applied to correct *p*-values for multiple tests. GO was further categorized into cellular components (CC), molecular functions (MF), and biological processes (BP), with a critical threshold set at FDR <0.05. Special attention was devoted to the top 5 KEGG pathways exhibiting high enrichment significance. The pathways with the most pronounced enrichment were meticulously elucidated, providing detailed insights into the molecular mechanisms and biological processes associated with the model genes. This comprehensive analysis contributes to a deeper understanding of the functional roles and pathways implicated in the context of atherosclerosis.

### 2.7 PPI network construction

The construction of the PPI network was facilitated through GeneMANIA (http://www.genemania.org), a platform designed for building PPI networks to predict gene function and identify genes with similar functions. The network integration algorithm employed various bioinformatics techniques, including site prediction, gene exchange, gene enrichment analysis, co-expression, co-localization, and physical interactions (38). Subsequently, the PPI network of model genes underwent analysis using GeneMANIA. KEGG and GO enrichment analyses were conducted on the network genes, leveraging the “clusterProfiler” R tool. The results of significant functional or pathway enrichment were visually represented in bubble plots, with corrections applied to achieve a threshold of *P* < 0.05. This analysis provides a comprehensive view of the functional relationships and enriched pathways within the PPI network of model genes, contributing valuable insights into the molecular interactions associated with atherosclerosis.

### 2.8 Exploration and validation of signature marker genes for atherosclerosis

To identify signature gene for atherosclerosis, further screening based on modular genes was conducted using Cox proportional—hazards regression model(COX regression) analysis on the training set data. Three machine learning techniques were employed to mitigate cohort bias. Supervised machine learning Support Vector Machine Recursive Feature Elimination (SVM-RFE) (39)was utilized for recursive classification of genes in the training set, and Least Absolute Shrinkage and Selection Operator regression(LASSO regression), implemented with the “glmnet” package in R (40), was employed to retain valuable variables. Sorting was performed using the “randomForest” package in R (41). Subsequently, the “mlr3verse” integrated software package facilitated the application of k-nearest, Naïve Bayes, Linear Discriminant Analysis, Logistic Regression, Recursive Partitioning, and Regression Trees as five machine learning algorithms. Evaluation of these algorithms was conducted through 5-fold and 10-fold cross-validation to compare model performance. The “pROC” function in the ROC R package (42) was employed to calculate the area under the curve (AUC) for the training and validation groups. This assessment aimed to gauge the performance of the model under different machine learning algorithms. The relationship between model genes and immunity was further explored using a CNN model. The main parameters and processes are as follows, (1) Feature Extraction:Feature extraction was performed using the deconvo_mcpcounter function from the IOBR package. This function provided a feature matrix based on gene expression characteristics of different cell types. Each sample's feature matrix was calculated and standardized, combining gene expression data with immune cell infiltration ratios for subsequent model training and testing. (2) CNN Architecture:We designed and trained a CNN to differentiate between atherosclerosis and control samples. The CNN architecture included: ① Input Layer: A 4D tensor with dimensions [length(genes),10,1]. ② First Convolutional Layer: 32 filters of size 3 × 3, ReLU activation, “same” padding. ③ Second Convolutional Layer: 16 filters of size 2 × 2, dilation rate (1,1), Softplus activation, “same” padding. ④ Pooling Layer: 2 × 2 max pooling. ⑤ Flattening Layer: Flattened multidimensional input to 1D. ⑥ Fully Connected Layer: 64 neurons, ReLU activation. ⑦ Dropout Layer: Dropout rate of 0.5 to prevent overfitting. ⑧ Output Layer: 1 neuron, Sigmoid activation for binary classification. The CNN model employed binary_crossentropy as the loss function, adam optimizer, and accuracy as the evaluation metric. The model was trained over 200 epochs. (3) CNN: The relationship between model genes and immunity was further explored using a CNN model. This involved creating a two-dimensional array by taking the quotient of model gene expression and immune cell infiltration. Subsequently, the CNN analysis was conducted, assessing performance through ROC analysis aimed to determine the effectiveness and value of the signature gene in clinical applications. (4) Multilayer Perceptron Architecture:For comparative purposes, we also designed and trained a multilayer perceptron (MLP). The MLP architecture included: ① Input Layer: A 1D tensor with dimensions length(genes) × 10 ② First Dense Layer: length(genes) × 10 neurons, ReLU activation, and a Dropout layer with a rate of 0.4. ③ Output Layer: 1 neuron, Sigmoid activation for binary classification.The MLP model also used binary_crossentropy as the loss function, adam optimizer, and accuracy as the evaluation metric, with 200 training epochs. (5) Model Evaluation:Model performance was evaluated using ROC curves and AUC values. ROC analysis was conducted on both training and testing sets for CNN and MLP models. Additionally, confusion matrices were generated to compare actual and predicted labels, and to assess classification performance at optimal thresholds.

### 2.9 Unsupervised clustering of patients with atherosclerosis

To classify the 69 disease group samples into different clusters based on the three atherosclerosis model genes, unsupervised clustering was conducted using the “ConsensusClusterPlus” R package (43). The process involved 1,000 cycles of k-means. The optimal number of clusters (k = 2) was determined through the cumulative distribution function (CDF), consensus matrix plot, and consistent clustering score. Additionally, kerning and scoring were performed using PCA and GSVA. This comprehensive approach facilitated the robust classification of patients with atherosclerosis into distinct clusters, providing insights into potential subtypes based on the expression patterns of the model genes.

### 2.10 Gene enrichment analysis and immune infiltration of model genes

To unravel the biological functions and signaling pathway importance of the model genes, Gene Set Enrichment Analysis (GSEA) was employed (44). Gene sets “h.all.v2023.2.Hs.symbols.gmt” and “c2.cp.kegg.gmt” from the MSigDB database (MSigDB, http://software.broadinstitute.org/gsea/MSigDB) (45) were utilized, focusing on “c2.cp.kegg.v11.0” gene sets. Pathways significantly enriched in the training set were identified based on the expression patterns of the signature gene. This analysis provides valuable insights into the biological functions and pathways implicated in atherosclerosis, shedding light on the potential molecular mechanisms driving the disease. Furthermore, an examination of immune infiltration in the context of model genes enhances our understanding of the interplay between these genes and the immune microenvironment in atherosclerosis.

### 2.11 Molecular docking

To analyze the binding affinity and interaction patterns of the drug candidates with the targets, the protein-ligand docking software AutodockVina 1.2.2 (46) was employed. Molecular structures of Dronabinol, Estradiol, Clofibrate, Nicotine, Zidovudine, and Indinavir were obtained from PubChem Compound Data (https://pubchem.ncbi.nlm.nih.gov/) (47). The 3D coordinates of CD36 (PDB ID: 4F7B), S100A10 (PDB ID: 1A4P), and CSNK1A1 (PDB ID: 6GZD) were retrieved from the Protein Data Bank (PDB) (http://www.rcsb.org/pdb/home/home.do). For the docking analysis, all protein and molecular files were converted to PDBQT format, excluding water molecules and adding polar hydrogen atoms. The grid box was positioned in the center, encompassing the region of each protein and allowing free molecular motion. The dimensions of the grid box were set to 126 Å × 126 Å × 126 Å, with a grid point distance of 0.05 nm. Molecular docking studies were executed using Autodock Vina 1.2.2 (http://autodock.scripps.edu/). This analysis provides valuable insights into the potential interactions between the drug candidates and the specified targets (CD36, S100A10, and CSNK1A1) at the molecular level.

### 2.12 Statistical analysis

Statistical analysis was conducted using R software version 4.3.1. One-way analysis of variance (ANOVA) and *t*-tests were employed to explore potential significant differences in atherosclerosis model genes, functional enrichment results, immune cell infiltration, and immune function scores among the patient groups. The significance level was set at *p*-values and false discovery rate (FDR) q-values below 0.05, indicating statistical significance. These analyses provide a rigorous statistical foundation for the interpretation of the study results and the identification of key factors associated with atherosclerosis.

## 3 Result

### 3.1 Single-cell dimensionality reduction swarming to obtain key subpopulations

**Figure 2:**
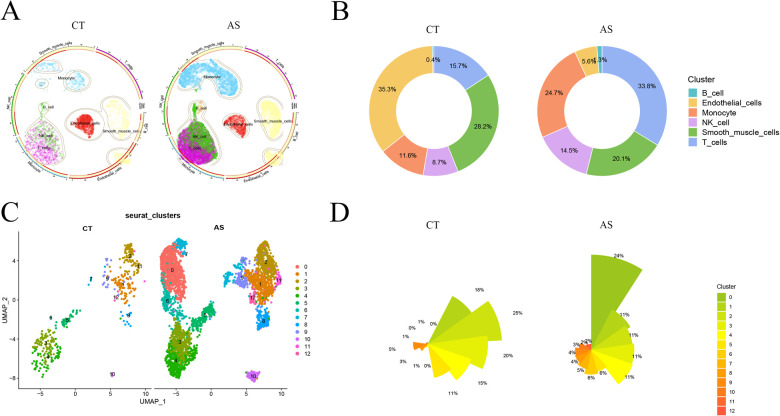
scRNA-Seqs data reduced to clusters.aS, atherosclerosis group. CT, control group; **(A)** UMAP cluster analysis of scRNA-Seq data. **(B)** scRNA-Seq various cell proportions; **(C)** UMAP reduced cluster analysis of monocyte subpopulations; **(D)** proportions of the number of small monocyte subpopulations.

To delve into the gene expression profile of atherosclerosis, our initial step involved normalizing and pre-processing the single-cell data from atherosclerotic samples (GSE159677) (Supplementary Figures 1A,B). We selectively retained single-cell data meeting the criteria of 200≤ nCount_ Features ≤2,500 and percent.mt <10. Subsequently, we employed UMI and gene correlation analysis to detect cells, achieving a correlation coefficient between nCount and nFeature (r = 0.92), indicating the qualification of cell quality (Supplementary Figure 1C). The RunPCA function in the Seurat software package was then utilized to perform PCA. We identified and mitigated potential batch effects in the original data, discovering anchors through PCA dimensionality-decreasing clustering (Supplementary Figure 1D). The top five ranked PC data were selected for further analysis (Supplementary Figures 1E,F). Using the findclusters function, we clustered the 17 subclusters into groups and annotated them using the “SingleR” package. This process identified six distinct cell classes: B cell, Endothelial cell, Monocyte, NK cell, Smooth muscle cell, and T cell. Visualization of normal and diseased samples was achieved through UMAP plots (Figure 2A). Notably, in this study sample, the proportions of Monocyte, T cell, and Endothelial cell populations in the diseased group were significantly higher compared to the normal group, as depicted in the scale graph (Figure 2B). Further analysis focused on the three cell types, with the data of the monocyte subpopulation exhibiting the most significant degree of change selected for subsequent exploration. Continuing our analysis, we focused on the monocyte data, downscaling it into clusters and presenting the results in UMAP plots (Figure 2C). This allowed for a detailed comparison of cellular changes in small subpopulations between the normal and disease groups. In this study sample, out of the 12 small monocyte subpopulations, subpopulations 0, 6, and 7 were notably more prevalent in the disease group (Figure 2D). This observation holds substantial value for our study, highlighting specific small monocyte subpopulations that may play a crucial role in the context of atherosclerosis.

### 3.2 Cellchat probes potential communication networks between cells

**Figure 3:**
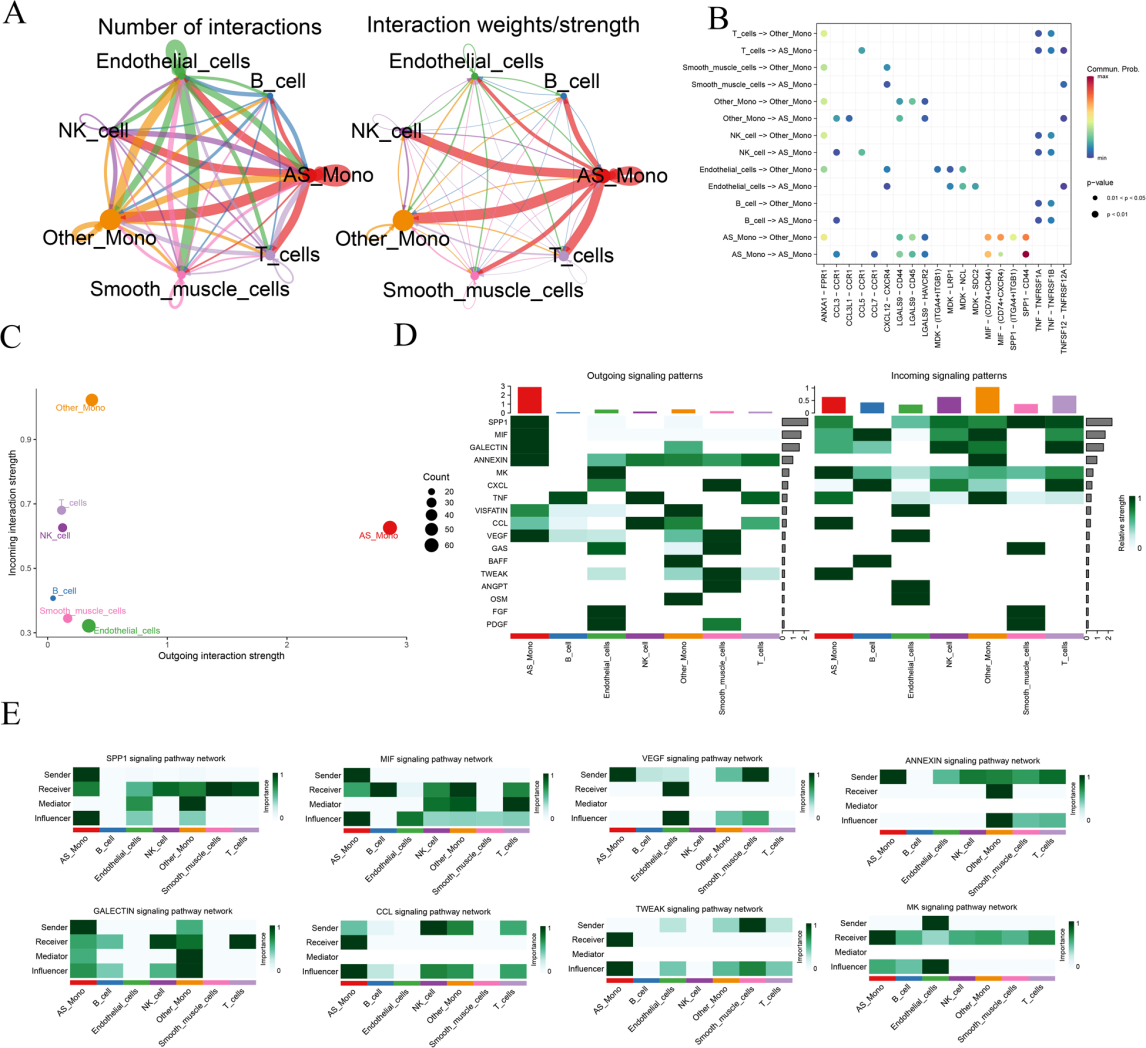
Analysis of cellular communication for monocyte data. AS_Mono, Differentially Highly Expressed Monocyte Subpopulations in Atherosclerotic Samples. Other_Mono, Other Monocyte Subpopulations Not Differently Expressed in Atherosclerotic and Normal Samples. **(A)** overview of the number of receptor-ligand pairs and strength of communication between different cell taxa communications; **(B)** receptor-ligand interaction relationships between different cell taxa communications; **(C)** statistics of the total output-input communication of cell taxa; **(D)** cell taxa-associated cytokine output-receipt relationships; and **(E)** relationships of the various types of cells to individual cytokines.

To further delve into the alterations occurring in monocytes within diseased tissues, we individually extracted monocyte data from the diseased and normal groups. Using the CellChat software package, we predictively analyzed the intercellular communication network. Initially, we quantified the number of receptor-ligand pairs and communication strength between different cell taxa (Figure 3A). Notably, the relevant communication of monocytes was significantly enhanced in the disease samples. Subsequently, utilizing bubble, scatter, and heatmap visualizations, we depicted the correlation between the types of cytokines released among cells and the quantity of signaling outputs and receptive contributions, respectively (Figures 3B–D). This analysis revealed that in the disease group, the release of Secreted Phosphoprotein 1 (SPP1), Macrophage Migration Inhibitory Factor (MIF) by monocytes, and the receptive communication of Galactoside—binding lectin (GALECTIN), Annexin family of proteins (ANNEXIN), and Vascular Endothelial Growth Factor (VEGF) were significantly enhanced. Additionally, the communication of C—C Motif Chemokine Ligand (CCL), Midkine (MK), and TNF—related Weak Inducer of Apoptosis (TWEAK) exhibited heightened activity. To provide a unified representation of the active cytokines, a heatmap was employed, showcasing the aforementioned dynamic cytokine interactions (Figure 3E). This comprehensive analysis sheds light on the intricate communication networks and altered cytokine dynamics involving monocytes in the context of atherosclerosis.

### 3.3 hdWGCNA access to core module genes

**Figure 4:**
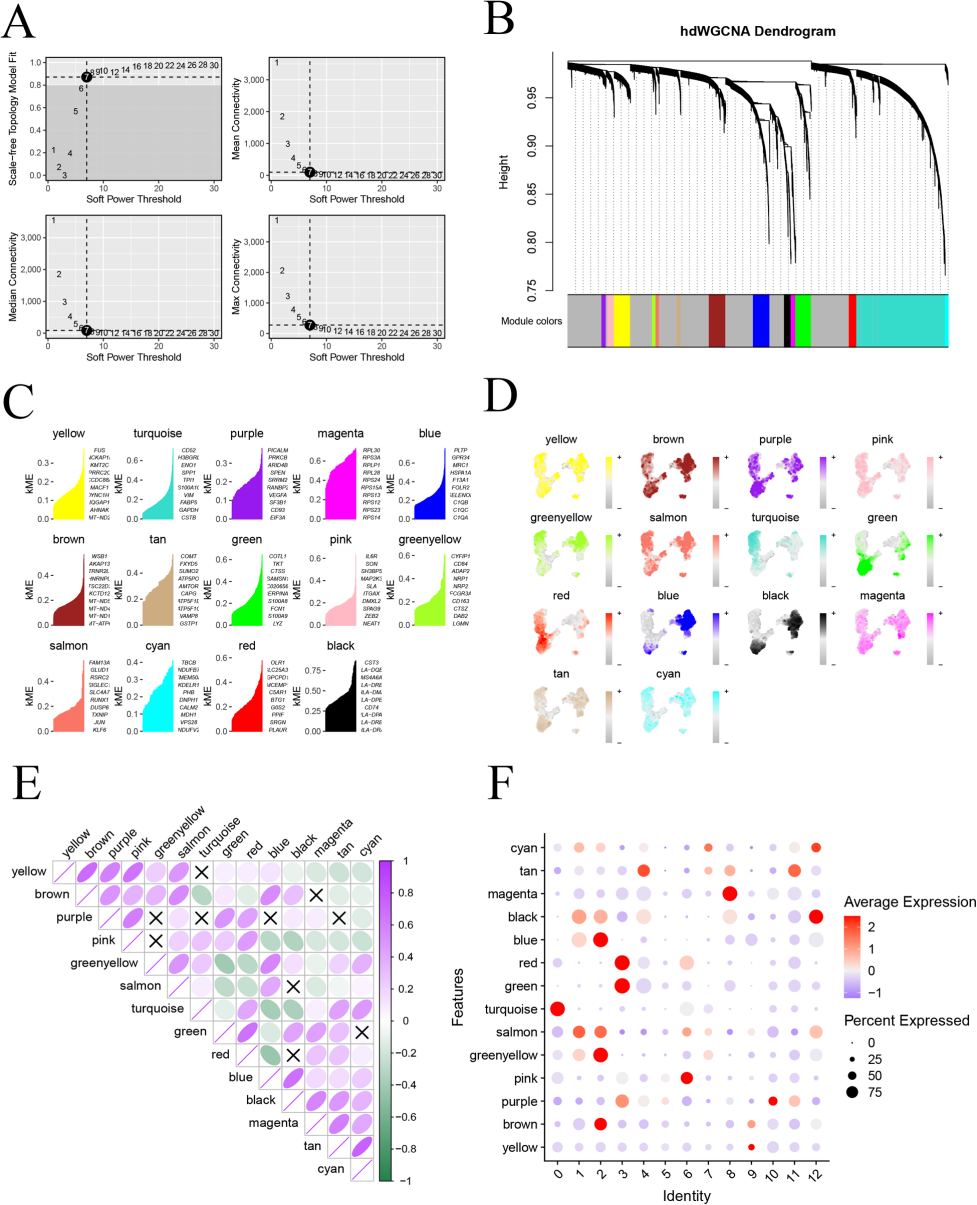
High-dimensional weighted co-expression network analysis; **(A)** soft threshold b = 7 and scale-free topological fit index; **(B)** clustering dendrogram for detecting combinatorially similar modules; **(C)** KEMs gene scoring for each module; **(D)** expression mapping map on UMAP for each module; **(E)** correlation analysis between modules; **(F)** correlation between modules and expression of small subpopulations of monocytes.

To delve deeper into the gene network associated with monocytes in atherosclerosis, we employed the “hdWGCNA” package to conduct hybrid dimensionally weighted gene co-expression network analysis. Initially, we utilized the “SetupForWGCNA” function to filter out genes expressed in at least 5% of the cells, excluding obvious abnormal data. Subsequently, the MetacellsByGroups function was employed for subcellular clustering of single-cell data. To calculate the soft threshold value, we used the “TestSoftPowers” function, setting it to 7 (Figure 4A), which provided the average connectivity for the subsequent construction of the co-expression network. The merged modules under the specified number of clusters were displayed (Figure 4B). Subsequently, we scored and visualized the genes under each module using the “UCell” package (Figure 4C). To illustrate the distribution of genes in each module on the UMAP graph, we mapped the scoring structure of each module into the color space of UMAP (Figure 4D). Additionally, exploring the relationship between each module was facilitated through a correlation heatmap (Figure 4E). In a final step, we used a bubble graph to depict the distribution of each module with monocyte subtypes. Notably, the most varied small subtypes of the monocyte disease group, specifically subtypes 0, 6, and 7, were predominantly located in the pink and turquoise modules (Figure 4F). This comprehensive analysis provides insights into the core module genes associated with distinct monocyte subtypes in the context of atherosclerosis.

### 3.4 Proposed time series analysis to study monocyte differentiation trajectories

**Figure 5:**
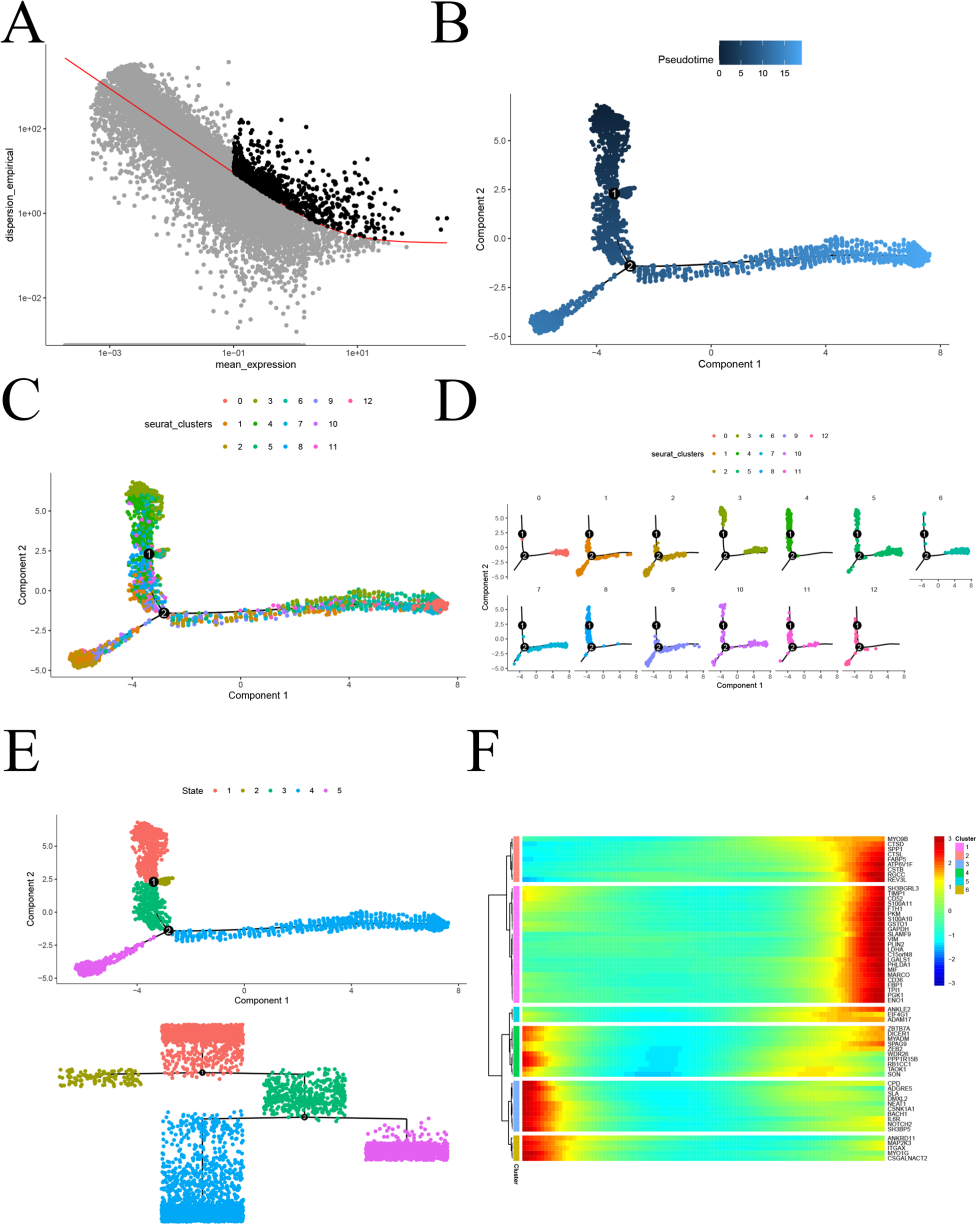
Proposed chronological analysis. **(A)** Screening of highly variable genes for atherosclerosis; **(B)** Proposed chronological measurement of the degree of cellular differentiation; **(C)** General overview of the 13 differentiation trajectories of the proposed chronological analysis; **(D)** Display of the 13 differentiation trajectories of the proposed chronological analysis, one by one; **(E)** Dendrogram State level proposed chronological analysis revealing the differentiation trajectories; **(F)** Heatmap of the expression of genes characteristic of atherosclerosis, arranged in chronological order.

To investigate the proposed chronology of monocyte differentiation trajectories, we utilized the “monocle2” package for analysis. Initially, we employed the dispersionTable function to calculate gene dispersion. Subsequently, we identified highly variable genes based on criteria such as average gene expression greater than or equal to 0.1 and empirical dispersion exceeding 1 times the fitted dispersion (Figure 5A). Using the plot_cell_trajectory function, we illustrated the proposed temporal differentiation pathway diagram. The color intensity reflects the progression of cell differentiation over time, with darker colors indicating earlier stages. The diagram reveals that monocyte differentiation progresses from the top down, moving from the middle towards the two sides of differentiation (Figure 5B). Furthermore, monocytes were classified into 11 key cell subtypes, and pseudotemporal analysis projected them for display (Figures 5C,D). To further confirm the specific direction of differentiation of monocyte subpopulations, an evolutionary tree diagram at the state level of monocytes was utilized (Figure 5E). Additionally, we performed a separate analysis of the pink and turquoise modules in hdWGCNA, which contained significantly altered genes. The analysis revealed that module genes were mainly differentiated from top to bottom and from the middle to the sides (Figure 5F). This comprehensive analysis provides valuable insights into the intricate differentiation trajectories of monocytes, shedding light on the temporal dynamics and specific directions of differentiation for distinct subpopulations in the context of atherosclerosis.

### 3.5 Immune cell infiltration analysis

**Figure 6:**
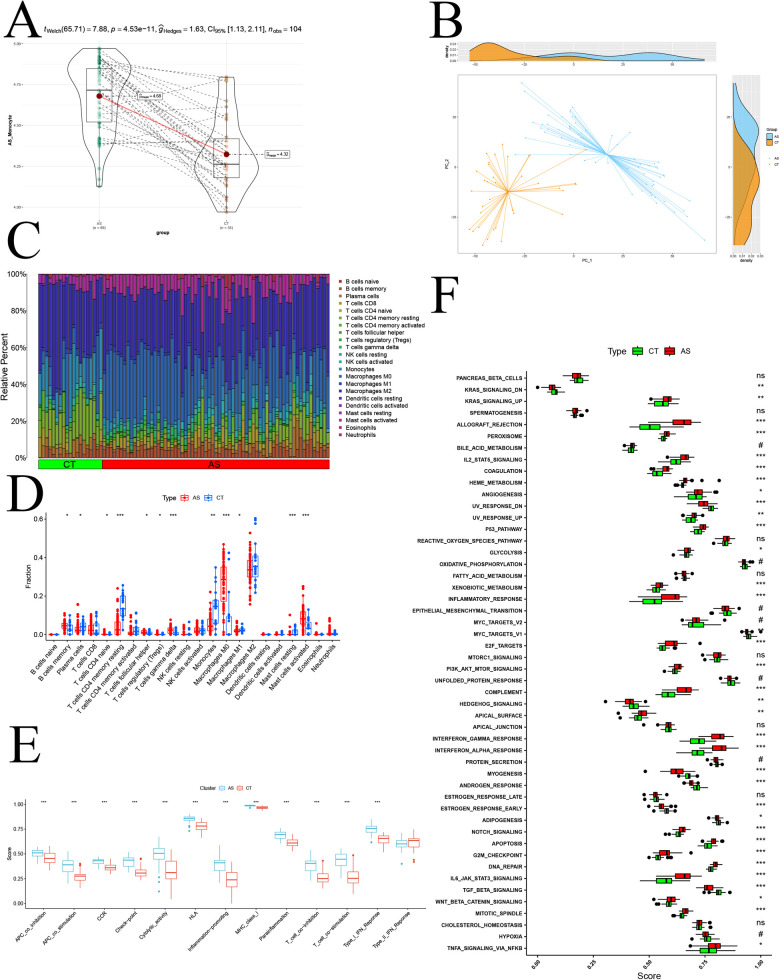
Bulk-level immune infiltration analysis. **(A)** GSVA disease-normal group difference analysis; **(B)** PCA analysis of disease-normal group samples; **(C)** proportion of various immune cell infiltration components; **(D)** immune cell infiltration difference analysis; **(E)** immune function scoring difference analysis; **(F)** normal-disease group ssGSEA difference analysis based on Hallmark gene sets.; *p* significance is indicated by “***” < 0.001, “**” < 0.01, “*” < 0.05.

To comprehensively validate the study of monocyte changes in atherosclerosis and assess overall immune infiltration, we transitioned to a transcriptomic atherosclerosis GEO dataset (GSE100927). This shift in perspective allowed us to measure gene expression at the level of the entire cell population (bulk). Before delving into the analysis, we preprocessed the GSE100927 and GSE43292 samples. Initially, we identified and mitigated batch effects between samples using box-and-line plots via the normalizeBetweenArrays function (Supplementary Figures 2A–D). Subsequently, we uncovered significant batch effects between the two datasets using PCA (Supplementary Figure 2E). Employing the ComBat function helped eliminate the batch effect (Supplementary Figure 2F). After the meticulous data processing, we proceeded to unveil the disparity in the activity of modular genes between samples in the disease and normal groups through GSVA analysis (Figure 6A). The observed *p*-value <0.01 indicated a significant difference at the entire bulk level. Further confirmation of the significant difference in the expression of modular genes between disease and normal groups was achieved through PCA (Figure 6B). Employing CIBERSORT, we compared 22 types of immune cells and depicted the differences in immune cell composition between the two groups using bar graphs (Figure 6C). Notably, in the disease group, activated Mast cells, Macrophages M0, B cells memory, and other immune cells exhibited high infiltration, while Plasma cells, Monocytes, and other immune cells showed low infiltration (Figure 6D). An immune function analysis was also conducted, revealing significant up-regulation in APC co-inhibition, APC co-stimulation, CCR, Checkpoint, Cytolytic activity, HLA, Inflammation Promoting, MHC class I, Parainflammation, T cell co-inhibition, T cell co-stimulation, and Type I-IFN Response (Figure 6E). Finally, leveraging the GSEA database of immunologic signature gene sets through the ssGSEA algorithm, we demonstrated the differences in immune infiltration between the disease and normal groups (Figure 6F). This comprehensive analysis provides a holistic view of the immune landscape in the context of atherosclerosis, highlighting specific immune cell populations and functional pathways implicated in the disease.

### 3.6 Module gene interaction analysis and enrichment analysis

**Figure 7:**
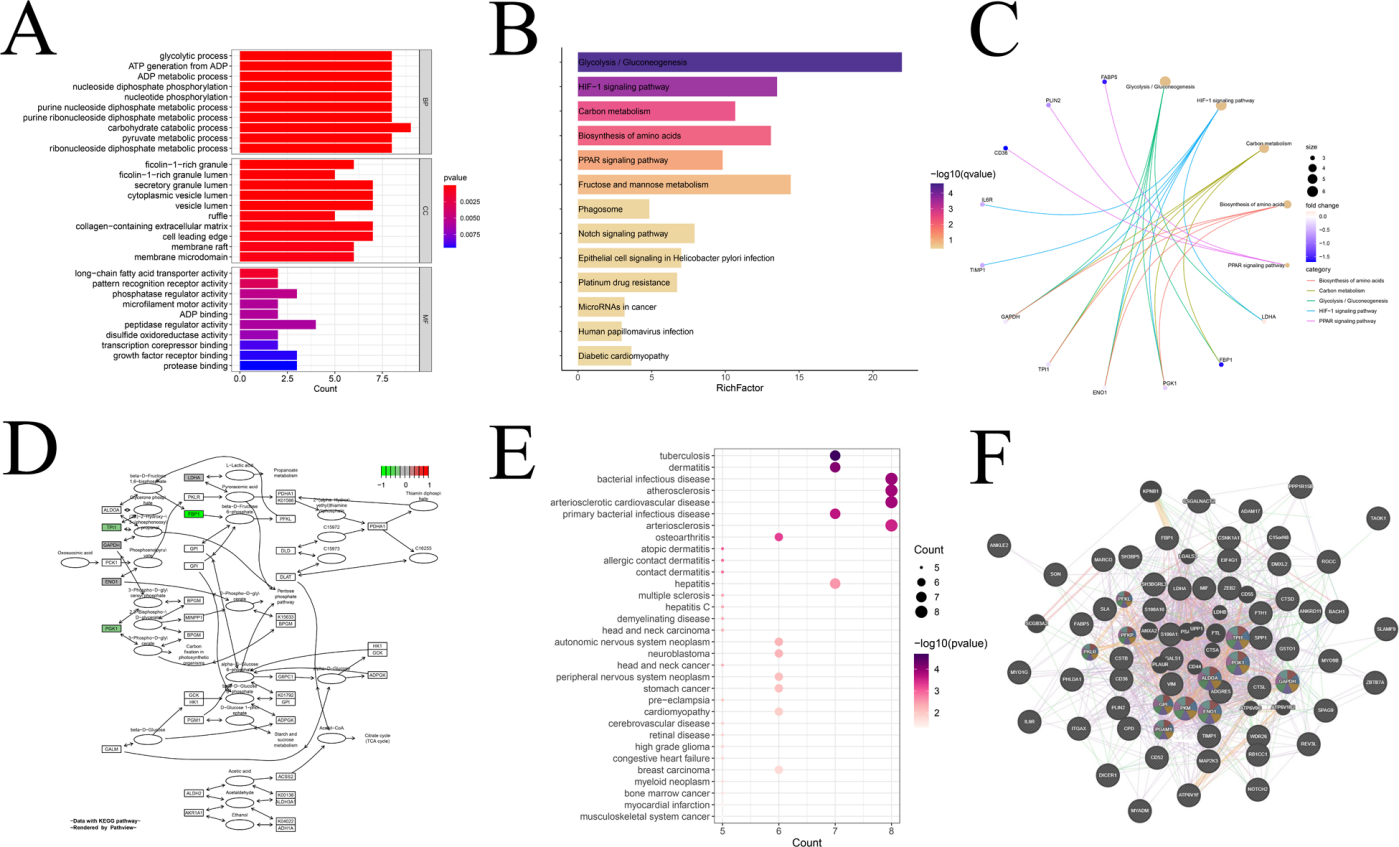
Interaction analysis and enrichment analysis of modular genes. **(A)** GO enrichment analysis of modular genes; **(B)** KEGG enrichment analysis of modular genes; **(C)** display of KEGG top five pathway-related genes; **(D)** display of KEGG enrichment of the most significant pathways; **(E)** DO enrichment analysis; and **(F)** Co-expression gene protein mutualistic network.

Furthermore, we conducted an in-depth analysis of the module genes obtained from hdWGCNA for relevant enrichment and selected significantly enriched items (q-value <0.05) for presentation. Beginning with the GO enrichment analysis, we delved into the characteristics of module genes in cellular components, biological processes, and molecular functions, presenting the top ten enriched pathways (Figure 7A). In the biological processes category, modular genes were prominently involved in biometabolism-related processes such as sugars, lipids, nucleotides, etc. The cellular components category demonstrated that modular genes concentrated in tissues related to cell membranes, vesicles, and exocrine granules. The molecular functions category reflected the regulatory role of modular genes in phosphorylation and dephosphorylation processes of various types of proteases, fatty acid transporters, and nucleotide binding-related molecules in the process of phosphorylation and dephosphorylation. This comprehensive enrichment analysis provides valuable insights into the functional roles and molecular pathways associated with the identified module genes, shedding light on their potential contributions to the complex processes underlying atherosclerosis. In addition, we conducted an evaluation of the biological activity and signaling functions through KEGG enrichment analysis (Figure 7B). This analysis not only reaffirmed the role of the module in glycolysis/gluconeogenesis, amino acid synthesis, and other pathways but also revealed enrichment in the HIF-1, PPAR, and Notch signaling pathways. The top five enriched pathways in KEGG were further highlighted (Figure 7C), and it was observed that the expression levels of all these genes were significantly lower than normal. Taking the most significantly enriched Glycolysis/Gluconeogenesis pathway as an example (Figure 7D), we demonstrated the inhibition of fructose-1,6-bisphosphatase, phosphoglycerate kinase 1, and triosephosphate isomerase 1. Additionally, we performed DO enrichment analysis (Figure 7E) and discovered that the modular genes were indeed most significantly enriched in atherosclerosis and its atherosclerotic heart disease. This multi-faceted enrichment analysis provides a nuanced understanding of the functional implications of module genes in the context of key biological processes and pathways associated with atherosclerosis. Finally, we elucidated the PPI network of the module genes by utilizing the GeneMANIA database (Figure 7F). Notably, nearly 80% of the proteins expressed by these genes exhibited co-expression relationships. Among them, 11 genes, namely GPI, PGK1, PFKL, ALDOA, TPI, PGAM1, PFKP, ENO1, PKM, GAPDH, and PKLR, were all implicated in sugar- and NADH-related metabolism, aligning with the enrichment results. This PPI network analysis enhances our understanding of the intricate relationships and functional collaborations among module genes, particularly in the context of metabolic pathways crucial to atherosclerosis.

### 3.7 Module gene-based machine learning screening and validation

**Figure 8:**
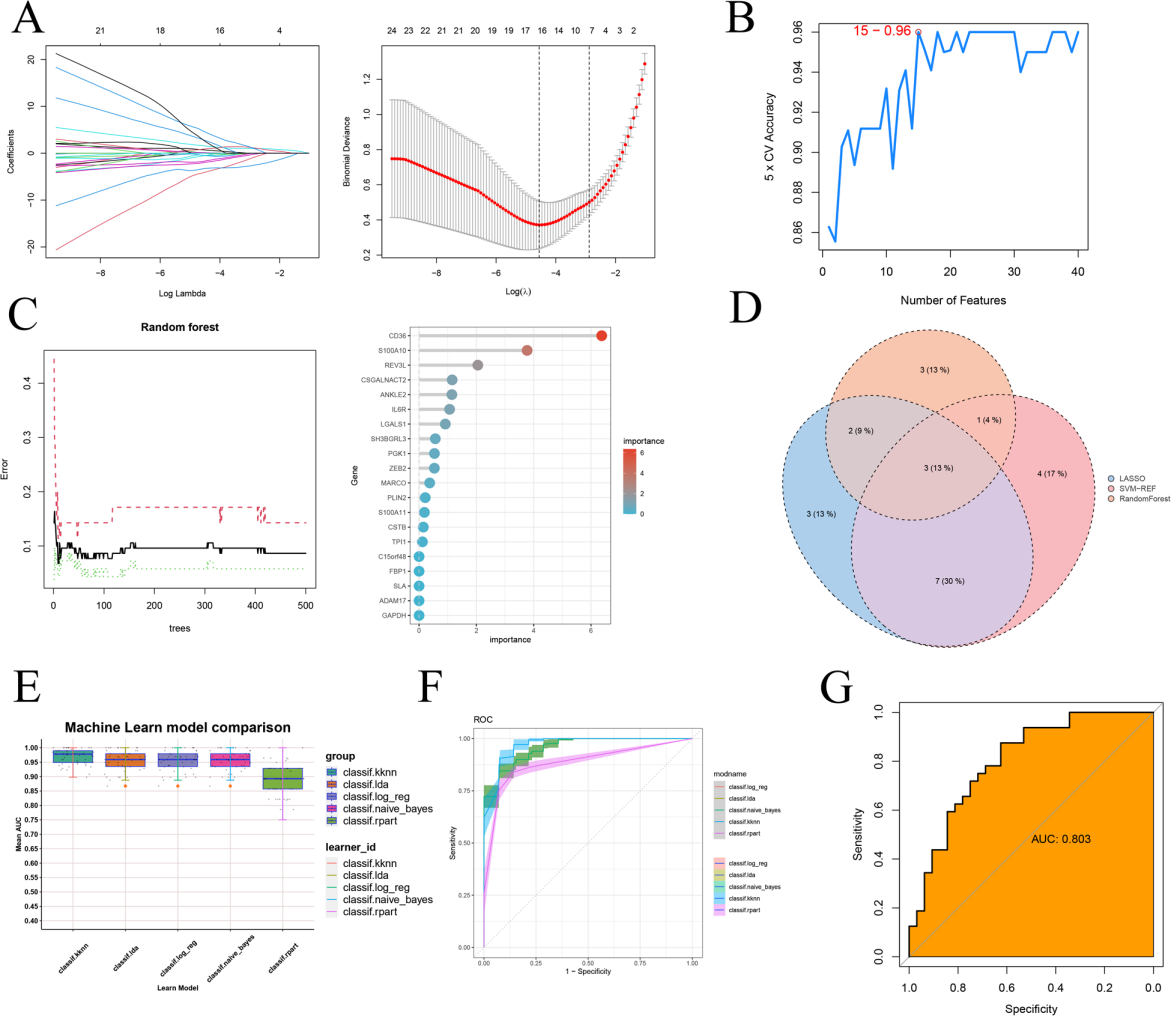
Machine learning screening sequencing feature genes and testing validation. **(A)** 10× cross-validation of LASSO algorithm tuning parameter selection, lasso coefficient distribution plot; **(B)** SVM-RFE validation of biometric gene expression; **(C)** Tree number vs. random forest error rate, lollipop graph showing gene importance ranking **(D)** Wayne's plot screening of three machine learning shared feature genes; **(E)** kknn, k-nearest. naïve_Bayes, Naïve Bayes. Ida, Linear Discriminant Analysis. log_reg, Logistic Regression. rpart, Recursive Partitioning and Regression Trees. Five additional ROC comparisons of feature genes for machine learning training sets; **(F)** Specific ROC demonstration for training sets of five machine learning algorithms; **(G)** ROC validation on external dataset.

Firstly, we employed three machine learning algorithms—LASSO, Random Forest, and SVM-RFE—to discern feature genes from the module genes and assess their diagnostic efficacy. For the LASSO algorithm, we identified the optimal λ, with an average error minimum of 0.01, through ten-by-ten cross-validation. Subsequently, the LASSO classifier was constructed based on this minimum λ value, revealing 15 feature genes (Figure 8A). Regarding the Support Vector Machine algorithm, the SVM-RFE approach achieved peak accuracy with 15 features. Consequently, by considering the collapsed average ranking order, 15 feature genes were identified (Figure 8B). In the case of Random Forest, we selected the 9 feature genes with the highest importance by amalgamating feature selection and classification tree results (Figure 8C). Subsequently, through the Venn diagram analysis, we identified three signature genes—CD36, S100A10, and CSNK1A1—as the intersection genes derived from the three machine learning algorithms (Figure 8D). Finally, we investigated the efficacy of the identified signature gene in effectively diagnosing and distinguishing the normal group from the atherosclerotic disease group. We assessed the performance of five machine learning algorithms—k-nearest, Naïve Bayes, Linear Discriminant Analysis, Logistic Regression, and Recursive Partitioning and Regression Trees—via 5-fold and 10-fold cross-validation (Figures 8E,F and Supplementary Table 1). The results indicated that the average AUC for these models consistently exceeded 0.85. Additionally, to mitigate the potential impact of chance in the sample results, we conducted external validation using another GEO transcriptome dataset (Supplementary Figure 3). Notably, we presented the evaluation results for the representative Linear Discriminant Analysis algorithm (Figure 8G), demonstrating an AUC exceeding 0.8.

### 3.8 CNN deep learning

**Figure 9:**
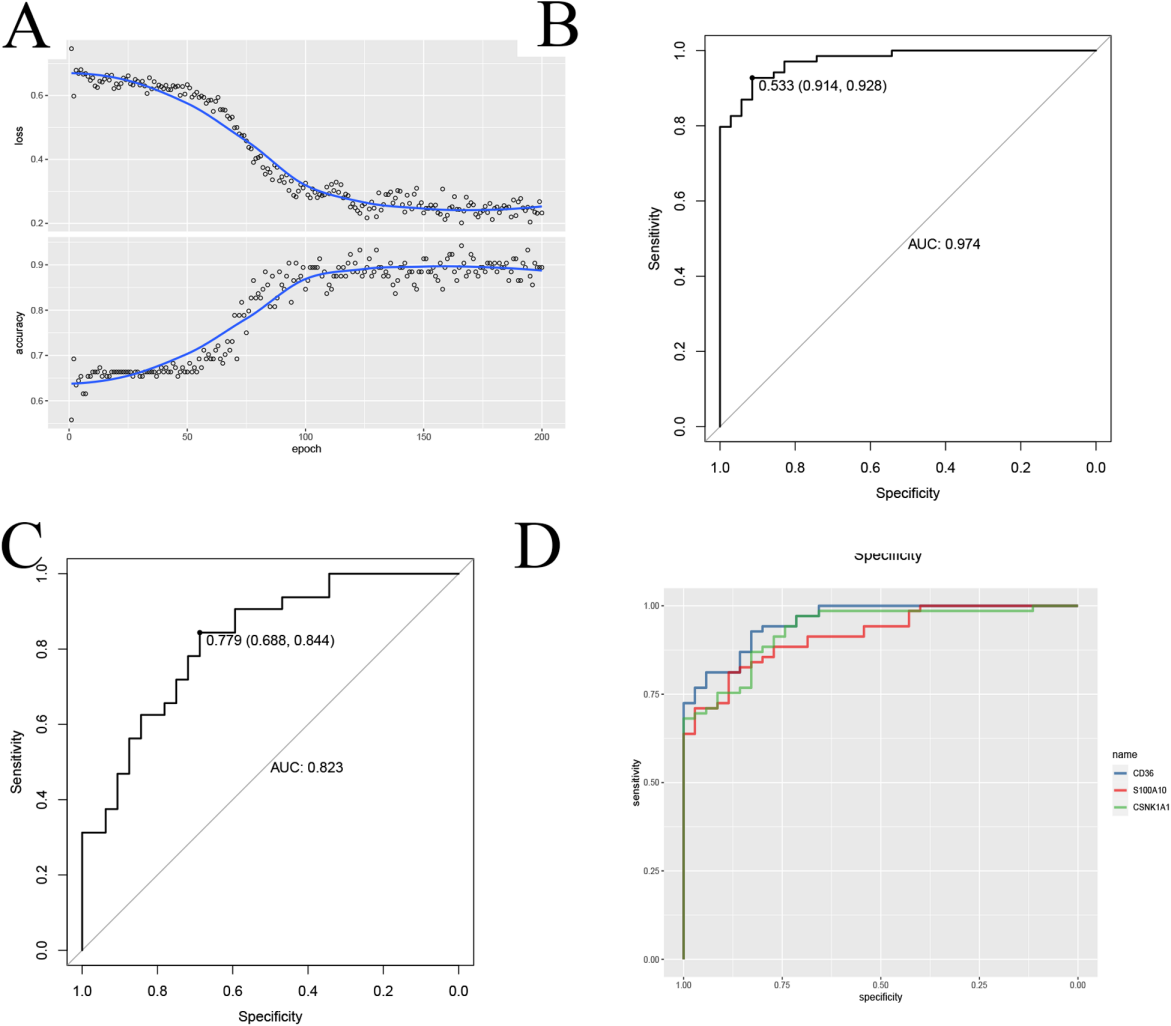
CNN to build diagnostic models and validate them. **(A)** CNN to validate feature genes; **(B,C)** CNN ROC training-validation group ROC demonstration; **(D)** Diagnostic performance ROC curves of the signature genes.

To further validate the Application value of the signature gene, we employed the CNN deep learning method. We constructed a two-dimensional array by juxtaposing the expression levels of the model genes with immune cell infiltration data. Subsequently, we utilized the CNN to train and validate the diagnostic accuracy of the model genes (Figure 9A). Remarkably, the accuracy rate reached approximately 0.9, and the AUC consistently exceeded 0.8 in both the training and validation groups (Figures 9B,C and Supplementary Table 2), providing robust evidence for the reliability of the signature gene diagnosis. To enhance the quantitative understanding of the diagnostic process involving the signature gene, we employed a logistic regression model to determine the applied regression coefficients, constructing a linear prediction model. The logistic regression model, incorporating the model genes, generated a column-line diagram illustrating atherosclerosis each gene received a score, and the cumulative score of the module genes reflected the risk of developing atherosclerosis. The ROC curves for each modeled gene were presented (Figure 9D). This suggests that the CNN model based on this signature gene provides more valuable insights into the patient's condition (Figure 9F).

### 3.9 Signature gene-based subtype construction and immune infiltration

**Figure 10:**
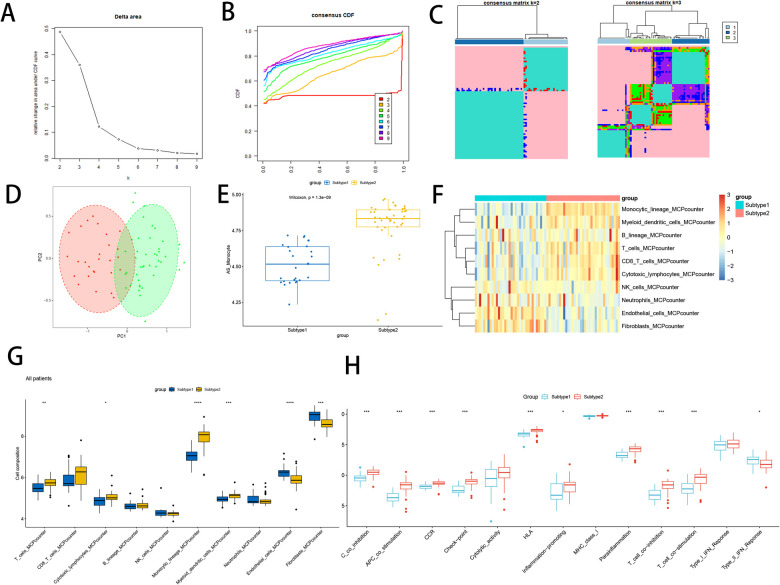
Constructs based on atherosclerosis subtypes. **(A)** Elbow method of Delta area to take the optimal k value; **(B)** Consensus CDF at k = 2–9; **(C)** Consensus matrix heat map at k = 2 and 3; **(D)** PCA analysis of two subtype samples; **(E)** Expression of two subtype samples assessed by GSVA; **(F)** Heat map of the difference in immune cell infiltration between subtypes; **(G)** Comparison of the difference in immune cell infiltration between subtypes; **(H)** Differential analysis of immune function between subtypes;. “****” indicates *P* < 0.0001.

This comprehensive analysis using signature gene of atherosclerotic monocytes resulted in the identification of two subtypes. By leveraging the elbow method, CDF plots, and consistency matrix heatmap, we determined the optimal grouping k = 2, 3 (Figures 10A–C). PCA and single-sample gene enrichment analysis (ssGSVA) showcased significant distinctions between the two subtypes (Figures 10D,E). Subtype 2 exhibited higher expression frequencies of modular genes, indicating its greater relevance in the disease group compared to subtype 1. Furthermore, heatmaps illustrated differences in modular genes and immune cell infiltration between the two subtypes (Figure 10F). Subtype 2 demonstrated pronounced expression levels of NK cells, lymphocytes, dendritic cells, and monocytes. Box-and-whisker plots displayed variations in immune cells between atherosclerosis-associated gene subtypes (Figure 10G), revealing significant increases in T lymphocytes, monocytes, and myeloid dendritic cells in subtype 2 relative to subtype 1. Conversely, endothelial cells and fibroblasts exhibited a decrease. Immune function analysis demonstrated that subtype 2 had a substantial increase in the proportion of APC co-inhibition, APC co-stimulation, CCR, Check point, HLA, Inflammation promoting, Parainflammation, T cell co-inhibition, and T cell co-stimulation, compared to subtype 1, while Type II-IFN Response function was down-regulated (Figure 10H).

### 3.10 Enrichment analysis of signature gene

**Figure 11:**
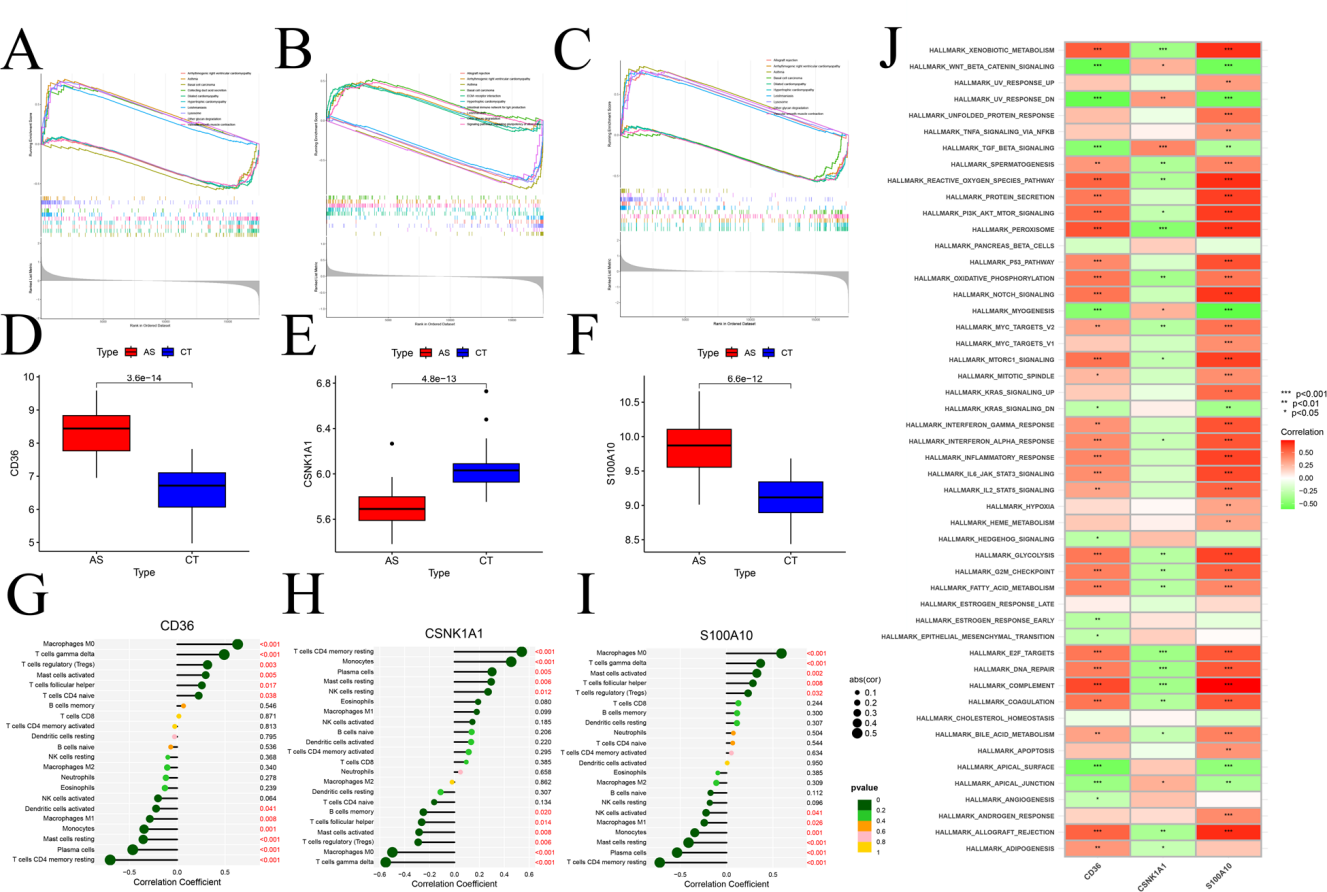
Enrichment, immunoassay analysis based on marker genes. **(A–C)** GSEA identification of signaling pathways associated with signature genes, **(A)** CD36, **(B)** CSNK1A1, **(C)** S100A10; – Box line plots of expression differences of modular genes, **(D)** CD36, **(E)** CSNK1A1, **(F)** S100A10; **(G–H)** immune cell infiltration analysis; **(G)** CD36, **(H)** CSNK1A1, **(I)** S100A10; **(J)** ssGSEA differential analysis of modular genes based on Hallmark gene sets.

GSEA analysis of signature gene provided valuable insights into their association with specific signaling pathways. CD36 (Figure 11A), CSNK1A1 (Figure 11B), and S100A10 (Figure 11C) exhibited significant correlations with various pathways. Notably, CD36 and S100A10 displayed negative correlations in Vascular smooth muscle contraction, Basal cell carcinoma, and different types of cardiomyopathy, and positive correlations in Allograft rejection and Asthma, among others. Conversely, CSNK1A1 showed opposite correlations. Comparing the expression changes of model genes in the normal and disease groups, both CD36 and S100A10 (Figures 11D,E) demonstrated elevated expression compared to the normal group, while CSNK1A1 (Figure 11F) exhibited decreased expression. In addition, an immune correlation analysis was conducted on the model genes, revealing a significant positive correlation between CD36 and S100A10 with Macrophage M0 and T cells-γ delt cells. Conversely, a significant negative correlation was observed with T cells CD4 memory resting, Monocytes, and Plasma cells cells (Figures 11G,H). Interestingly, these correlations stand in stark contrast to the CSNK1A1 correlation pattern (Figure 11I). Moreover, ssGSEA analysis of model genes in the marker gene set of the GSEA database (Figure 11J) revealed that CD36 and S100A10's activities in the respective marker gene sets were precisely opposite to CSNK1A1, highlighting distinct regulatory roles of these genes in the context of atherosclerosis.

### 3.11 Molecular docking based on model genes to explore their potential therapeutic targets

**Figure 12:**
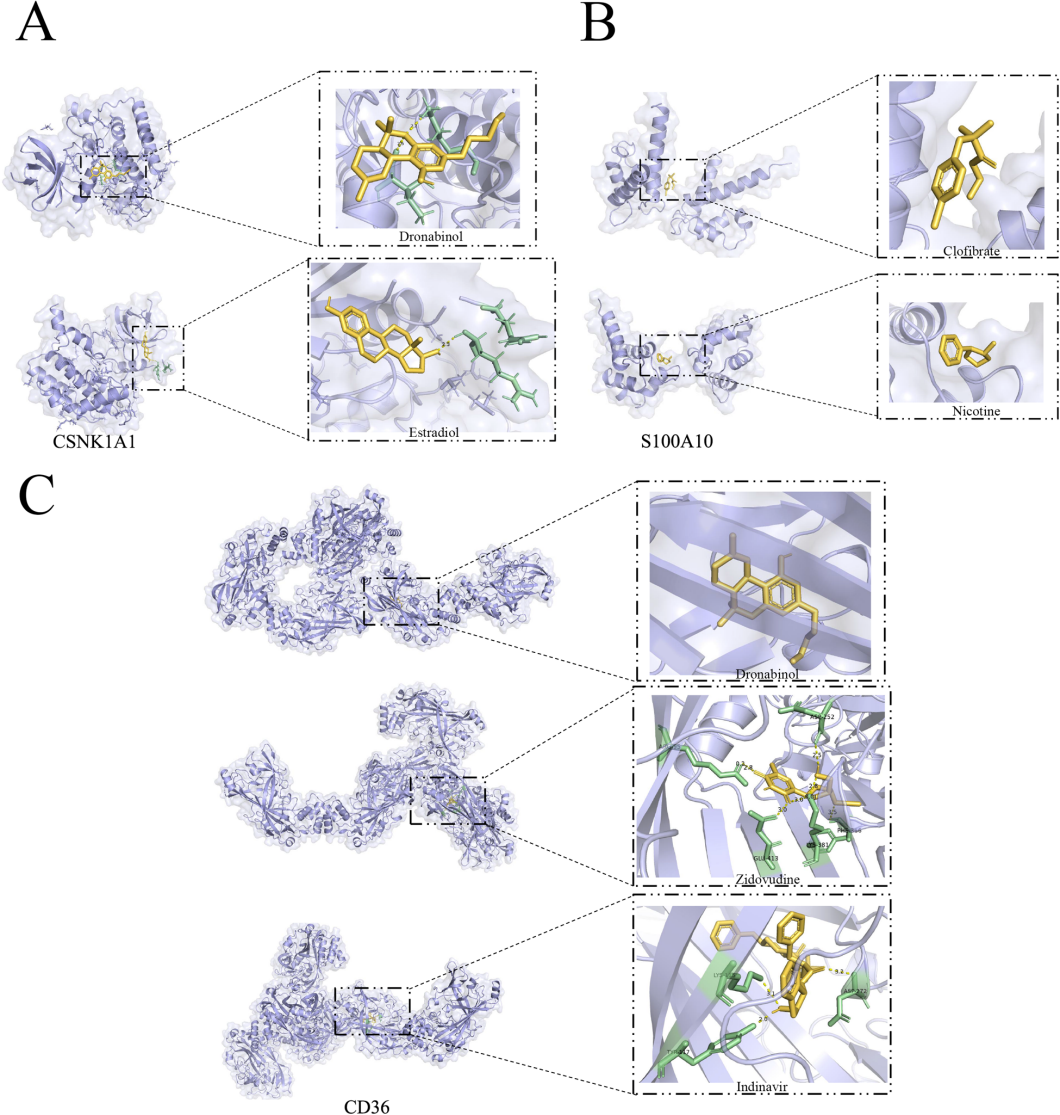
Marker gene-based docking of receptor proteins with ligand molecules. **(A)** CSNK1A1 docked with small molecules Dronabinol and Estradiol; **(B)** S100A10 docked with small molecules Clofibrate and Nicotine; **(C)** CD36 docked with Dronabinol and Zidovudine, Indinavir binding mode. (Yellow: Ligand; Green: macromolecule docking target structure).

Molecular docking analysis using Autodock Vina provided valuable insights into the binding poses and interactions of the drug candidates with their respective protein targets. The results indicated that the drug candidates formed visible hydrogen bonding and strong electrostatic interactions with the proteins, suggesting a potential for effective binding and therapeutic impact in the context of atherosclerosis. The generated binding energies further contribute to understanding the strength of these interactions, aiding in the assessment of drug efficacy. It's fascinating to see how these drug candidates, Dronabinol and Estradiol, effectively occupied the hydrophobic pocket of the CSNK1A1 target (Figure 12A), demonstrating promising binding energies. The fact that both drugs exhibited binding energies lower than −5 kcal/mol and formed at least 1–2 hydrogen bonds with adjacent proteins is indicative of strong binding activity.This underscores their potential as effective therapeutic agents for atherosclerosis by interacting with the specific protein target. It's intriguing to observe the differences in binding energies and interactions between drug candidates and their respective protein targets. While Clofibrate and Nicotine showed binding energies around −4.25 kcal/mol for S100A10 (Figure 12B), the absence of hydrogen bonds in the protein conformation suggests that their binding activity might be limited. Understanding the nuances of these interactions is crucial for evaluating their potential clinical utility. The molecular docking results for CD36 are particularly promising. The binding energies of Dronabinol, Zidovudine, and Indinavir are all below −7 kcal/mol (Figure 12C), indicating robust binding activity. Additionally, the formation of more than two hydrogen bonds in the binding conformation of CD36 with Zidovudine and Indinavir further supports their strong interaction, making them potential ideal drug candidates. This molecular docking analysis provides valuable insights into the potential efficacy of these drugs in targeting CD36.

## 4 Discussion

Identification of atherosclerosis is pivotal for proactive intervention, aiming to alleviate the socioeconomic burden of atherosclerotic cardiovascular diseases (ASCVD) (48, 49). Uncovering potential susceptibility markers and elucidating their underlying mechanisms represent effective strategies for predictive diagnosis and targeted prevention. Our investigation highlights CD36, S100A10, and CSNK1A1 as pivotal features in atherosclerosis, showcasing their collective prowess In terms of diagnosis, prognosis, and therapeutic performanceEmploying single-cell sequencing, we conducted a thorough bioinformatics analysis to integrate novel modular genes emerging in the atherosclerosis microenvironment via high-dimensional weighted gene co-expression network analysis, focusing on monocytes within the diseased microenvironment. Subsequently, we utilized three machine learning approaches (Random Forest, LASSO, and SVM-RFE) to identify core atherosclerotic features. Validation was performed using logistic regression, linear discriminant analysis, Naïve Bayes, k-nearest neighbor, and decision tree models. A CNN model was constructed to elucidate the correlation between core features and immune infiltration. The entire process harnessed multiple machine learning algorithms to elucidate the fundamental signature genes intrinsic to atherosclerosis, ultimately pinpointing CD36, S100A10, and CSNK1A1 as genetically correlated markers. The diagnostic performances of these markers were validated using ROC analysis, achieving an AUC exceeding 80%. Furthermore, our study delves into the segregation of two atherosclerosis subtypes, enhancing the precision of immune profile differentiation and facilitating tailored treatment strategies through systematically clustered patient samples.

Atherosclerosis is a chronic, lipid-driven inflammatory condition involving multiple pathways. While monocyte-derived data are crucial for identifying targets, their role within the complex pathophysiology of atherosclerosis warrants thorough and critical examination. Previous studies have shown that ARID5B gene expression is regulated by DNA methylation and involved in dysregulating lipid metabolism and inflammatory pathways through monocyte transcriptome and epigenome analyses (50). These findings reveal potential genetic changes in atherosclerotic monocytes/macrophages and highlight the importance of lipid metabolism pathways and inflammation in atherosclerotic plaque formation, a view that aligns with our current study. Our functional enrichment analysis of emerging modular genes in the atherosclerotic microenvironment, particularly through KEGG analysis, revealed significant enrichment in the peroxisome proliferator-activated receptor (PPAR) pathway, closely linked to lipid metabolism. PPARγ, primarily present in adipose tissue and the immune system, is crucial for adipocyte differentiation, cholesterol metabolism, and inflammatory responses. The development of atherosclerosis is characterized by elevated levels of LDL and very low-density lipoprotein (vLDL), along with reduced levels of high-density lipoprotein (HDL) in patients with hyperlipidemia and diabetes mellitus. One critical and intricate area of research focuses on regulating the electronegativity of LDL to mitigate the risk of atherosclerosis. Untargeted lipidomic analysis has shown that lipid profiles vary between subgroups with lower LDL electronegativity (L1) and higher LDL electronegativity (L5). The top 10 lipid species enriched in L1 were independently linked to fatal events within one year. These findings indicate that reduced LDL electronegativity is associated with changes in the LDL lipidome, suggesting it may be a novel risk factor for adverse outcomes in patients with acute coronary syndrome (ACS) (51). Moreover, increased electronegativity of LDL is associated with its pro-inflammatory and pro-apoptotic properties. These electronegative LDL [LDL(-)] are more prevalent in patients with high cardiovascular risk, serving as biomarkers to monitor atherosclerosis progression (52). Among the factors influencing LDL electronegativity, cytokines such as IL-4 and IL-13 enhance the ability of monocytes to oxidize LDL, whereas IFN-γ inhibits this process (53). Additionally, reducing LDL oxidation and increasing its electronegativity can be achieved with antioxidants like vitamins E and C, potentially slowing the progression of atherosclerosis in dialysis patients (54).

In addition to its role in lipid metabolism and inflammation, PPARγ is also closely related to CD36, a core gene among the marker genes we are currently investigating, further underscoring its significance in atherosclerosis. CD36 functions as a receptor for scavenging oxidized low-density lipoprotein (oxLDL) and aids in removing cholesterol deposits from arterial walls, thereby mitigating atherogenesis. However, it is important to note that the CD36-PPARγ pathway also plays a role in the early stages of atherosclerosis development. The activation of the CD36-PPARγ pathway not only fosters cholesterol efflux but also augments the synthesis of HDL and facilitates reverse cholesterol transport, leading to a reduction in atherosclerotic burden. Furthermore, this pathway exerts inhibitory effects on cholesterol synthesis within hepatocytes, curtailing *de novo* cholesterol production by downregulating the activity of 3-hydroxy-3-methylglutaryl coenzyme A reductase (HMGR). These regulatory actions hold therapeutic significance in the context of atherosclerosis (55, 56).

In summary, the pivotal involvement of the CD36-PPARγ pathway in atherosclerosis underscores its significance in both the prevention and treatment of atherosclerosis and its concomitant metabolic syndrome. This pathway intricately modulates lipid metabolism, cholesterol transport, and inflammatory responses, offering multifaceted therapeutic avenues for addressing atherosclerotic conditions.

In parallel with marker gene the classification of patients into C1 and C2 subtypes using three signature genes aims to enhance patient differentiation for more precise treatment. The intricate connection between atherosclerosis, immune inflammation, and tumorigenesis (57, 58) underscores the significance of investigating immune infiltration and immune function scores in patients with these subtypes, accomplished through the utilization of ssGSEA. At the level of immune cell infiltration, the C2 subtype exhibited significant overexpression, particularly in T cells, the Monocytic lineage, Cytotoxic lymphocytes, and Myeloid dendritic cells. These cell types are actively involved in the inflammatory process, with a primary focus on pathogen removal during the inflammatory response and the maintenance of a stable immune environment in the body. Conversely, the C1 subtype, characterized by high expression in Endothelial cells and Fibroblasts, plays a crucial role in maintaining normal physiological status. It contributes to regulating vascular response, immune cell migration, and tissue repair to cope with tissue damage during inflammation.

At the immune function level, elevated levels of both inflammatory and anti-inflammatory responses in C2 may be the primary contributors to persistent or chronic inflammation. This dual response pattern aligns with the concept of quasi-inflammation, where transient inflammation serves as a host defense mechanism, while prolonged inflammation is associated with detrimental tissue changes observed in conditions such as diabetic retinopathy (DR) and other age-related disorders (59). In addition to elevated levels of inflammation and anti-inflammatory antagonism, C2 also exhibits significant para-inflammation. Quasi-inflammation, characterized by increased expression of the anti-inflammatory cytokine IL-10 and a shift in macrophage plasticity from pro-inflammatory M1 to anti-inflammatory M2 polarization, serves as a mediator between basal inflammation and intense inflammatory responses (60). While mature inflammatory responses are typically associated with infection or tissue damage, quasi-inflammatory responses in C2 indicate tissue reactions to deleterious stress induced by various stressors, such as oxidative stress, hyperglycemia, or hypercholesterolemia. This complements the diverse risk factors and underlying etiologies previously discussed, collectively influencing the development of atherosclerotic disease. Meanwhile, C1 exhibits a stronger response to type II interferon than C2, suggesting that C1 activates inflammatory cells during inflammation, releasing IFN-γ to enhance their ability to combat bacteria and viruses. Additionally, C1 prompts antigen-presenting cells to efficiently present antigens, thereby regulating and enhancing immune system activity in C2.

In summary, C2 represents a “classical” activation state primarily triggered by infection and inflammation. These cells demonstrate robust microbicidal activity and produce inflammatory mediators crucial for clearing pathogens and diseased cells. However, excessive activation may lead to tissue damage. On the other hand, C1 represents an “alternative” activation state involved in inflammation regulation, tissue repair promotion, and immune homeostasis regulation.

Our signature genes, S100A10/p11, and CSNK1A1, are intricately linked to the development of various diseases and cancers. In our targeted approach towards the CD36 receptor, molecular docking was conducted to investigate potential therapeutic agents. Notably, CD36 exhibits robust binding activity with Indinavir and Zidovudine. Zidovudine (AZT), a pyrimidine synthetic analog, has been clinically utilized in the past for treating HIV-1 and preventing mother-to-child transmission. Specifically, Zidovudine belongs to the class of nucleoside reverse transcriptase inhibitors (NRTIs) (61). Zidovudine has proven efficacy in reducing the incidence of opportunistic infections and tumors, while also increasing the count of helper T lymphocytes (62). Additionally, Indinavir, another drug initially employed in the treatment of HIV/AIDS, functions as a protease inhibitor within highly active antiretroviral therapy. Notably, Indinavir has exhibited activity against the protease of human T lymphotropic virus type 1 (HTLV-1). There is ongoing exploration of novel medical applications of Indinavir, including its potential in preventing or treating conditions such as obesity, type II diabetes mellitus, diabetic nephropathy, and nonalcoholic fatty liver disease. In summary, the antiretroviral drugs Indinavir and Zidovudine exhibit promise beyond their established use. Our investigation, utilizing data from the Comparative Toxicogenomics Database (https://ctdbase.org/), reveals their significant inhibitory effect on the overexpression of CD36. This inhibition, in turn, impedes the macrophage phagocytosis of ox-LDL, a process integral to the formation of foam cells and the progression of atherosclerosis. These findings suggest a broader potential for the therapeutic application of Indinavir and Zidovudine beyond their original scope in treating HIV, extending into the realm of atherosclerosis management.

The intriguing discovery in our study indicates that Dronabinol binds to both CD36 and CSNK1A1 targets, exhibiting robust molecular bioactivity and suggesting a potential role in atherogenesis. Notably, Tetrahydrocannabinol (Δ9-THC) has been demonstrated to activate cannabinoid receptor 1 (CB1), triggering inflammation and oxidative stress in vascular endothelial cells. This could represent a plausible avenue through which cannabis may impact cardiovascular health. In a model employing human stem cell-induced vascular endothelial cells, genistein demonstrated the capacity to counteract the oxidative stress and inflammatory reactions initiated by Δ9-THC. This suggests that genistein's ability to inhibit CB1 receptors might antagonize the effects of Δ9-THC, potentially mitigating the development of atherosclerosis induced by Δ 9-tetrahydrocannabinol (63). Consequently, targeting tetrahydrocannabinol could emerge as a promising therapeutic strategy for atherosclerosis treatment (64).

Despite the innovative approach and significant findings, this study has several limitations that need to be addressed. Firstly, the limited sample size may affect the generalizability and reproducibility of the results. Although single-cell sequencing provides high-resolution data, the small number of samples analyzed could lead to biased outcomes, reducing the reliability of the identified biomarkers. Secondly, focusing on monocytes might overlook the contributions of other critical cell types in atherosclerosis. As a complex disease involving various cellular interactions, concentrating solely on monocytes may not provide a comprehensive understanding of its mechanisms. Thirdly, the complexity of the machine learning models used, including Random Forest, LASSO, SVM-RFE, and the CNN, presents challenges for interpretation and reproducibility. While these models enhance predictive accuracy, their intricate nature can hinder straightforward replication and clear interpretation of results. Lastly, although molecular docking based on CD36, S100A10, and CSNK1A1 has been used to predict targeted therapies for guiding clinical treatment, these predictions have not yet been experimentally validated. While the discussed therapeutic drugs may positively impact modeled gene expression and contribute to the recovery of atherosclerosis patients, these findings need validation through extensive clinical trials. Experimental confirmation is crucial to determine the broader impacts and clinical applicability of these molecular targets.Addressing these limitations in future studies will be essential for validating and expanding upon the current findings, ultimately contributing to a deeper understanding and more effective management of atherosclerosis.

## 5 Conclusion

In summary, our study highlights monocyte infiltration as a crucial contributor to atherosclerosis development, with CD36, S100A10, and CSNK1A1 emerging as prominent biomarkers for disease detection. These results furnish a robust scientific foundation for advancing the diagnosis and treatment strategies for individuals afflicted with atherosclerosis.

## Data Availability

The original contributions presented in the study are included in the article/Supplementary Material, further inquiries can be directed to the corresponding authors.

## Supplementary material

Supplementary Figure S1 Quality control of scRNA-seq data **(A)** Expression of scRNA-seq data before quality control. **(A)** Each point corresponds to one cell. nFeature_RNA represents the number of genes expressed in the cell, nCount_RNA indicates the number of UMIs expressed in the lower dial, and percent.mt represents the proportion of UMIs of mitochondrial genes in the cell to the total UMIs in each cell; **(B)** Expression of scRNA-seq data after quality control; **(C)** UMIs vs. scatter plot of total number of genes; **(D)** PCA down to cluster analysis before de-batching; **(E)** elbow drop plot of PCA; **(F)** PCA down to cluster analysis after de-batching.

Supplementary Figure S2 Quality control of transcriptome data. **(A)** boxplot of intergroup batch effects before de-batching of GSE100927; **(B)** boxplot of intergroup relationships after de-batching of GSE100927; **(C)** boxplot of intergroup batch effects before de-batching of GSE43292; **(D)** boxplot of intergroup relationships after de-batching of GSE43292; **(E)** boxplots of intergroup relationships before de-batching of GSE100927 vs. GSE43292 dataset batch effect PCA plots; **(F)** PCA plots showing the relationship between GSE100927 and GSE43292 datasets after de-batching.

Supplementary Figure S3 Evaluation results of ROC test for machine learning in the validation group. **(A)** Linear Discriminant Analysis; **(B)** Logistic Regression; **(C)** Recursive Partitioning and Regression Trees; **(D)** Naïve Bayes **(E)** k-nearest.

Supplementary Table S1 Machine Learning evaluation data on core genes.

Supplementary Table S2 CNN Scoring to evaluate data on core genes.
