# Supplementary figures and images for: Multi-modal transcriptomics: integrating machine learning and convolutional neural networks to identify immune biomarkers in atherosclerosis
Source: Front Cardiovasc Med. 2024 Nov 26;11:1397407. doi: 10.3389/fcvm.2024.1397407 (PMC11628520; doi:10.3389/fcvm.2024.1397407)

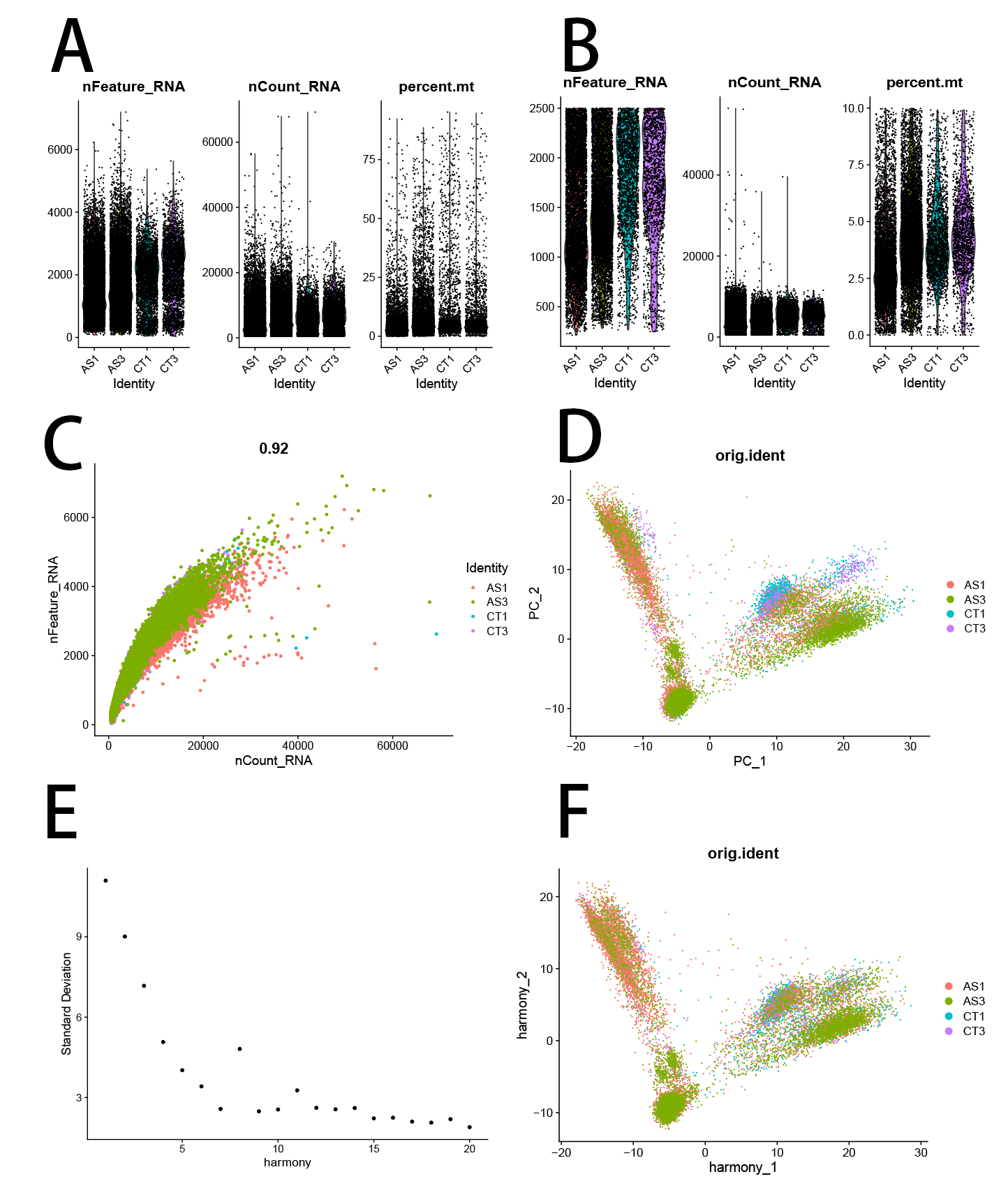

Supplement: Supplementary Figure S1 [file Image1.tif]

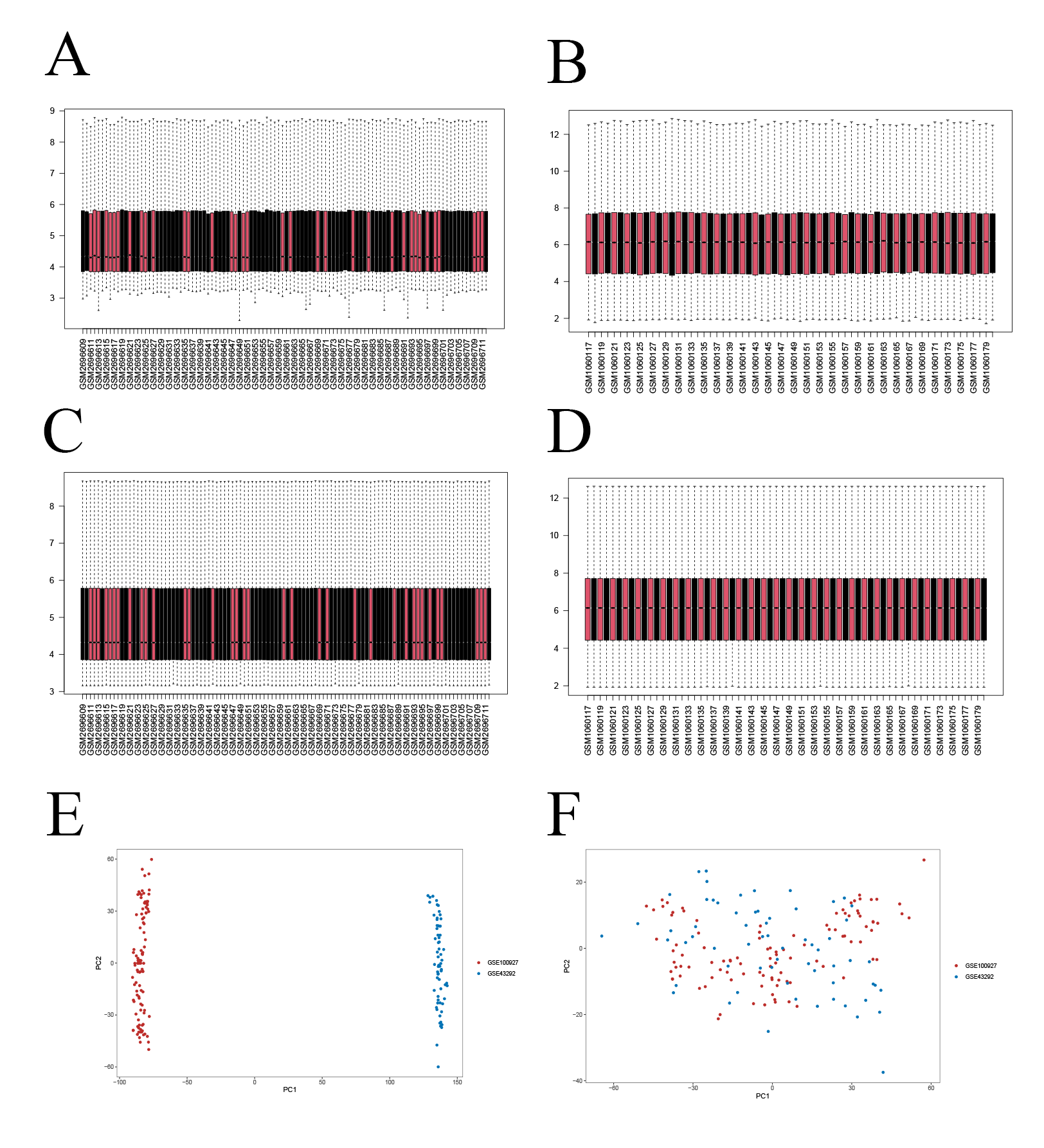

Supplement: Supplementary Figure S2 [file Image2.tif]

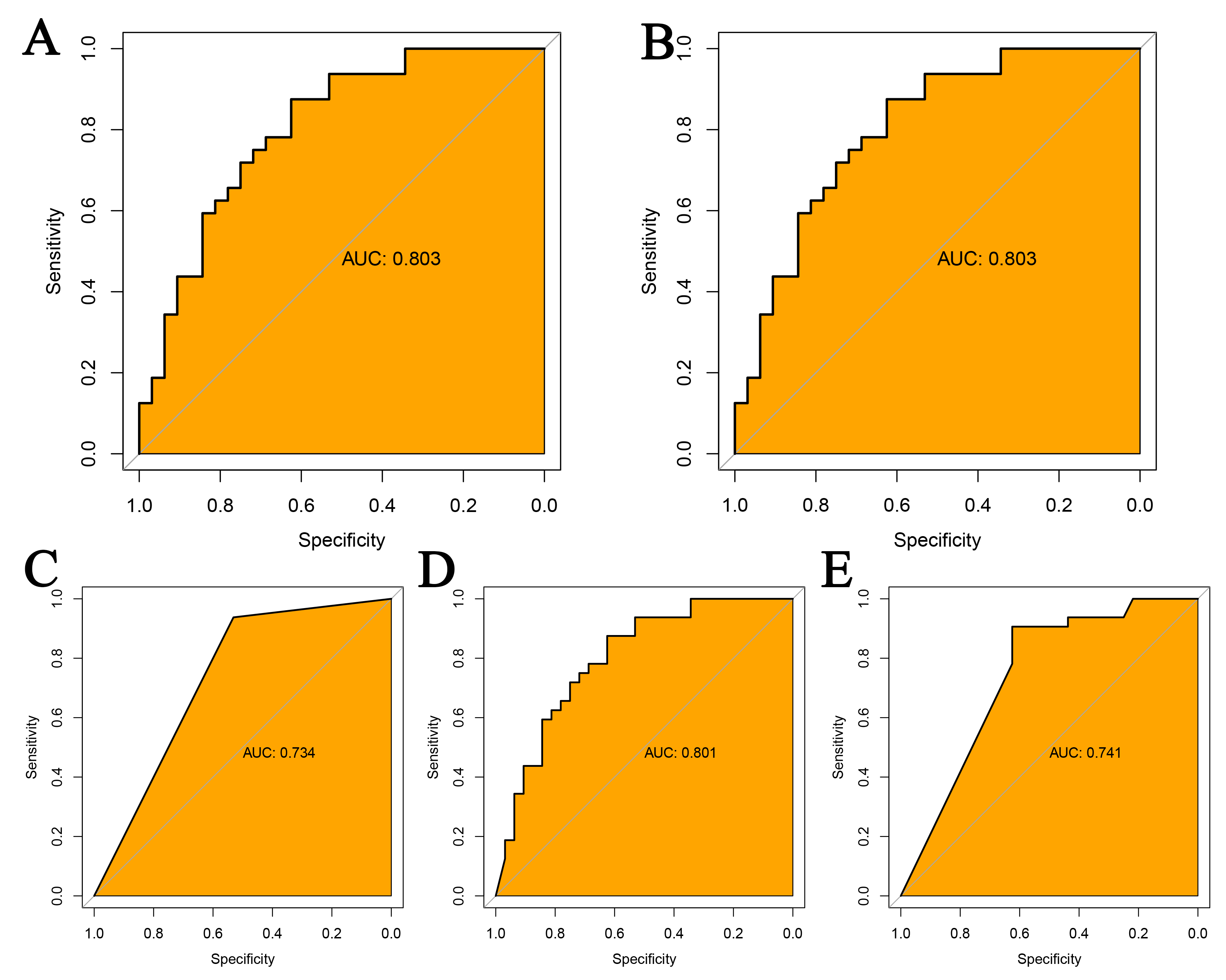

Supplement: Supplementary Figure S3 [file Image3.tif]
